# Heterophyllin B ameliorates diabetic lower limb ischemia by inhibiting SMOX to activate the Nrf2 antioxidant pathway

**DOI:** 10.1186/s13020-026-01440-x

**Published:** 2026-06-10

**Authors:** Shudong Lin, Yunchao Huang, Yiqiong Wang, Yuhui Xue, Shuyuan Lou, Qiuru Wang, Yaoting Wang, Yunyun Lu, Jiangyue Wu, Shujing Zhang, Houfa Yin, Ting Zhang, Lan Li, Ling Zhang

**Affiliations:** 1 School of Life Sciences, Zhejiang Chinese Medical University, Hangzhou, 310053 Zhejiang China; 2 School of Medical Technology and Information Engineering, Zhejiang Chinese Medical University, Hangzhou, 310053 Zhejiang China; 3 School of Pharmaceutical Science, Zhejiang Chinese Medical University, Hangzhou, 310053 China; 4 College of Pharmaceutical Sciences, Zhejiang University, Hangzhou, 310058 China; 5 Innovation Institute for Artificial Intelligence in Medicine, Zhejiang University, Hangzhou, 310018 China; 6 Eye Center, The Second Affiliated Hospital, School of Medicine, Zhejiang University, Hangzhou, 310009 China; 7 Zhejiang Provincial Key Laboratory of Ophthalmology, Zhejiang Provincial Clinical Research Center for Eye Diseases, Zhejiang Provincial Engineering Institute On Eye Diseases, Hangzhou, 310009 China

**Keywords:** Diabetic lower limb ischemia, Heterophyllin B, Spermine oxidase (SMOX), Nrf2, Oxidative stress, Endothelial dysfunction

## Abstract

**Background:**

Diabetic lower limb ischemia (DLLI) is a serious complication of diabetes with limited therapeutic options. Heterophyllin B (HET-B), a bioactive cyclopeptide from *Pseudostellaria heterophylla*, possesses antioxidant properties. However, its therapeutic mechanism in DLLI remains unclear.

**Purpose:**

This study aims to investigate the protective effects of HET-B against DLLI and elucidate the underlying metabolic and molecular mechanisms.

**Methods:**

A murine DLLI model was established in streptozotocin-induced diabetic mice via femoral artery ligation. Therapeutic efficacy was assessed by laser Doppler imaging, histopathology, and immunofluorescence. A high glucose-induced endothelial injury model was established using HUVECs. Endothelial function was evaluated by tube formation, migration, and wound healing assays. Single-cell RNA sequencing data (GSE165816) were analyzed to identify key metabolic targets. Mechanistic validation was performed using Spermine oxidase (SMOX) gene silencing and pharmacological activation with spermine.

**Results:**

HET-B significantly improved hindlimb blood flow recovery, promoted angiogenesis with increased CD31, α-SMA, VEGF, and eNOS, and attenuated inflammation with reduced TNF-α, IL-6, IL-1β, and TGF-β in ischemic muscles. In HUVECs, HET-B restored high glucose-impaired endothelial function, promoted Nrf2 nuclear translocation with NQO1 upregulation, suppressed TNF-α expression, and subsequently reduced Caspase-1/3 activation. Bioinformatic analysis identified SMOX as a key dysregulated gene in diabetic endothelium, and HET-B reversed its overexpression both in vitro and in vivo. Immunofluorescence co-staining confirmed SMOX and Nrf2 localization in CD31-positive endothelial cells, with HET-B reversing SMOX upregulation while restoring Nrf2 activation. SMOX knockdown mimicked HET-B effects, whereas SMOX activation with spermine abrogated HET-B-mediated protection, Nrf2 activation, and NF-κB suppression, confirming that HET-B acts through functional inhibition of SMOX.

**Conclusion:**

HET-B alleviates DLLI by inhibiting SMOX to activate Nrf2-mediated antioxidant defense and suppress inflammatory signaling, suggesting that the SMOX-Nrf2 axis may represent a potential therapeutic target for DLLI.

**Supplementary Information:**

The online version contains supplementary material available at 10.1186/s13020-026-01440-x.

## Introduction

Diabetes mellitus constitutes a pervasive global health crisis, with its macrovascular complications accounting for substantial morbidity and mortality [1, 2]. Among these, diabetic lower limb ischemia (DLLI) represents a particularly devastating outcome, often progressing to critical limb-threatening ischemia and amputation [3]. Current Western pharmacological management, primarily relying on antiplatelet agents, statins, and vasodilators, provides only symptomatic relief and fails to reverse the underlying pathology [4]. These conventional regimens are further limited by their narrow targeting, inability to rectify the core metabolic disturbances, and associated risks such as bleeding and drug resistance [5, 6]. This profound therapeutic inadequacy underscores the urgent need for novel treatment strategies that target the fundamental drivers of DLLI.

The complex pathogenesis of DLLI is fueled by a self-perpetuating cycle of hyperglycemia-induced metabolic disturbance, relentless oxidative stress, and chronic inflammation [7, 8]. Sustained hyperglycemia promotes mitochondrial dysfunction and an overproduction of reactive oxygen species (ROS), which disrupts the cellular redox balance and is a principal instigator of endothelial dysfunction [9]. This oxidative burden, in turn, activates key pro-inflammatory signaling pathways, most notably the NF-κB cascade, leading to the upregulated expression of pivotal inflammatory cytokines such as TNF-α and IL-6 [10, 11]. The transcription factor nuclear factor erythroid 2-related factor 2 (Nrf2) and its downstream effector NAD(P)H quinone oxidoreductase 1 (NQO1) constitute an essential endogenous defense mechanism against this damage [12]. By orchestrating a comprehensive antioxidant response, the Nrf2 pathway is vital for maintaining endothelial homeostasis and breaking the vicious cycle linking oxidative stress and inflammation [13].

In traditional Chinese medicine (TCM), *Pseudostellaria heterophylla* (Tai Zi Shen) is traditionally used to replenish *Qi* and nourish *Yin*. From a modern mechanistic perspective, *Qi-Yin* deficiency is closely linked to impaired antioxidant capacity and heightened oxidative stress [14]. This pattern of deficiency is widely recognized as a core pathogenic basis for diabetes-related complications, particularly those involving microvascular damage and impaired tissue repair. Heterophyllin B (HET-B), a major cyclopeptide isolated from *Pseudostellaria heterophylla*, has been reported to possess potent antioxidative and anti-inflammatory properties [15]. Given its multi-target pharmacological profile, which simultaneously addresses multiple pathological facets, HET-B represents a compelling candidate for further investigation in the context of DLLI.

Based on the preceding evidence, this study was designed to investigate the protective effects of HET-B against DLLI and to elucidate the underlying mechanisms. We first examined whether HET-B could improve limb perfusion and reduce inflammation in a murine DLLI model, and then used a high glucose‑induced endothelial injury model to explore the cellular mechanisms. By mining a public single‑cell RNA‑sequencing dataset (GSE165816) from diabetic foot ulcers, we identified SMOX as a candidate upstream target and experimentally tested the hypothesis that HET-B acts by inhibiting SMOX, thereby activating the Nrf2‑mediated antioxidant pathway and suppressing inflammatory and apoptotic cascades.

## Material and methods

### Establishment of the DLLI mouse model and HET-B treatment

Twenty-four healthy male C57BL/6 J mice (20–25 g) were purchased from Shanghai SLAC Laboratory Animal Co., Ltd. All mice were maintained under specific pathogen-free (SPF) conditions in the Experimental Animal Center of Zhejiang Chinese Medical University (12‑h light/dark cycle, 22 ± 1 °C, 55 ± 5% humidity, free access to food and water).

Type 1 diabetes was induced via intraperitoneal administration of streptozotocin (STZ) (CAS: 18883-66-4, Sangon Biotech, China) dissolved in citrate buffer (pH 4.5). Following daily 8 h fasts, mice received STZ (55 mg/kg) for five consecutive days. Two weeks after the initial injection, diabetic mice were defined as those exhibiting random blood glucose levels > 16.5 mM. For DLLI induction, successfully modeled diabetic mice were anesthetized with an intraperitoneal injection of 3% sodium pentobarbital (50 mg/kg). The left hindlimb was shaved and disinfected with iodine and 75% ethanol. A longitudinal skin incision of approximately 1 cm was made along the femoral artery trajectory. The fascia was bluntly dissected to expose the femoral neurovascular bundle. At the proximal site to the deep femoral artery bifurcation, the femoral artery and vein were double ligated with 8–0 polypropylene sutures, followed by severance of the femoral artery to ensure complete disruption of blood flow. This procedure is referred to as femoral artery ligation (FAL) [16]. The successfully modeled mice were randomly divided into four groups (n = 6 per group): the model group, the low-dose HET-B group (HET-B-L, 3 mg/kg), the high-dose HET-B group (HET-B-H, 6 mg/kg) [17]. The normal and model control groups received saline daily by oral gavage, while the HET-B groups received the corresponding concentrations of HET-B (CAS: 145459-19-4, Chengdu Efa Biotechnology, China) at a volume of 0.1 mL/10 g body weight. Drug administration continued for an additional 21 days post-surgery [18].

All animal procedures were approved by the Institutional Animal Care and Use Committee of Zhejiang Chinese Medical University (Approval No. IACUC-20230731-07) and conducted in accordance with the NIH Guide for the Care and Use of Laboratory Animals.

### Laser Doppler imaging analysis

Laser Doppler imaging technology, coupled with a laser Doppler perfusion imager (Perimed AB, Sweden), was employed for dynamic tracking of lower limb blood flow changes across all experimental groups. Detection was performed at the preoperative baseline and postoperative days 0, 3, 7, and 14. For statistical analysis, the ratio of the average perfusion volume at the ischemic site to that at the normal site was calculated.

### Immunofluorescence staining of tissues

Muscle tissues were fixed in 4% paraformaldehyde, dehydrated through a graded sucrose series, embedded in paraffin, and sectioned at a thickness of 5 μm. Following deparaffinization and rehydration, antigen retrieval was carried out using sodium citrate buffer. The sections were then permeabilized with 0.5% Triton X-100 (BS084, Biosharp, China) and blocked with 3% BSA (ST025, Beyotime, China). Subsequently, they were incubated overnight at 4 °C with primary antibodies targeting CD31 (1:200, ER31219, HUABIO, China), α-SMA (1:2000, ET1607-53, HUABIO, China), VEGF (1:200, ER30607, HUABIO, China), eNOS (1:200, AF0096, Affinity, China), Angiopoietin-2 (1:200, 31499-1-AP, Proteintech, USA), TNF-α (1:300, 60291-1-Ig, Proteintech, China), IL-6 (1:100, R1412-2, HUABIO, China), IL-1β (1:100, HA601002, HUABIO, China), TGF-β (1:300, 21898-1-AP, Proteintech, China), NF-κB (1:200, 80979-1-RR, Proteintech, China), and, SMOX (1:1000, 35842 T, CST, USA) followed by a 1-h incubation with appropriate secondary antibodies at room temperature. The sections were further incubated with TSA dyes (Servicebio: iF488-Tyramide, G1231; iF594-Tyramide, G1242) for 10 min, and nuclei were stained with DAPI for 10 min. Finally, the samples were mounted with an anti-fade mounting medium (P0126, Beyotime, China). Images were captured using a digital slide scanner (VS120-S6-W, Japan) and analyzed accordingly.

### Cell culture

HUVECs (Human Umbilical Vein Endothelial Cells) were obtained from the China Center for Type Culture Collection (CCTCC) and cultured in Dulbecco's Modified Eagle Medium (DMEM; C11885500BT, Gibco, Germany) containing 5.5 mM D-glucose and 10% fetal bovine serum (FBS; C04001, VivaCell, China) at 37 °C and 5% CO₂. For SMOX activation experiments, cells were co-treated with spermine (SP, 10 μM, MCE, HY-B1777A) under high glucose (HG) conditions [19]. Following the manufacturer's instructions, cells were transfected with SMOX siRNA (siSMOX, 50 nM, genOFF h-SMOX_2500A, Ribobio).

### Cell viability

HUVECs were categorized into a normal group and a HG injury model group. In the HG injury model group, glucose solutions at varying concentrations (25, 33.3, 50, 75 and 100 mM) were administered, while the normal group received an equivalent volume of basic culture medium (FBS-free). Cell viability was assessed using the Cell Counting Kit-8 (CCK-8, Beyotime, China) to determine the glucose concentration at which HG-induced cellular damage occurs. Subsequently, HUVECs cells were exposed to different concentrations of HET-B (0, 1, 5, 10, 20, 50, 75 and 100 µM) to evaluate its effects on cell viability using the CCK-8 assay. Finally, the cells were divided into three experimental groups: Control, HG, and HG + HET-B. The HG + HET-B group was treated with HET-B at the same concentration range (0, 1, 5, 10, 20, 50, 75 and 100 µM). The optimal concentration of HET-B was determined based on cell viability measurements obtained from the CCK-8 assay.

### Tube formation

Matrigel (CAS: 119,978–18-6, Corning, USA) was allowed to polymerize in 24-well plates. HUVECs were then plated at a density of 3 × 10^4^ cells per well and treated with one of the following media: control group (NG, LG DMEM medium), the model group (HG, 44.5 mM d-glucose + LG DMEM medium), the mannitol group (Aladdin, 69–65–8) (MN, 44.5 mM mannitol + LG DMEM medium), and the administration group (HG + HET-B, HG + 10 μM HET-B). After 4 h of incubation, tube formation was visualized and imaged. Tube length and number of tubes were quantified using ImageJ software.

### Transwell

The migration capacity of cells was evaluated using the Transwell assay. Logarithmic-phase cells were pre-treated for 48 h as previously described, then re-seeded into the inserts of 24-well plates (Cat. No. 725301, Nest, China). Following 24 h of incubation, the inserts were carefully removed. Cells that had migrated to the bottom of each well were fixed with 4% paraformaldehyde and subsequently stained with 1% crystal violet (Cat. No. C805209, Macklin, China). Random microscopic fields were imaged using a fluorescence inverted microscope live cell imaging system (Zeiss Axio Observer. A1, Germany). The number of migrated cells was quantified using ImageJ software (Version 1.49, USA).

### Wound healing assay

The wound healing assay was adopted to evaluate cell proliferation capacity. Logarithmic-phase cells were seeded into 6-well plates (703,001, Nest, China) and incubated for 24 h until full confluency was achieved. A sterile 200 μl pipette tip was used to create scratches in the cell monolayers, after which PBS (C3580, VivaCell, China) washes were performed to eliminate detached cells. Cells were divided into groups as previously described. For each cell group, cell migration was monitored and imaged at 0 h, 12 h, 24 h, and 48 h using a fluorescence inverted microscope-based live cell imaging system. Subsequently, the scratch area (cell-free region) was quantified via the Wound Healing Size Tool plugin of Image-J software.

### Assessment of oxidative stress

To assess oxidative stress, cells were first incubated with DCFH-DA (5 μM, S0035S, Beyotime, China) for 30 min to detect ROS and superoxide; fluorescence (excitation wavelength: 488 nm) was visualized using a fluorescence microscope (Zeiss, Zeiss Axio Observer. A1) and quantified via ImageJ.

### Cellular immunofluorescence

Cells in the logarithmic growth phase were seeded onto sterile coverslips (80346-0910, Shitai, China) pre-positioned in 24-well plates and grouped as described. Following 48 h of culture, cells were fixed with 4% paraformaldehyde, permeabilized with 0.5% Triton X-100 (BS084, Biosharp, China), and blocked with 3% BSA (ST025, Beyotime, China). The samples were then incubated overnight at 4 °C with primary antibodies against SMOX and Nrf2 (1:200, 12721 T, CST, USA), followed by a 1-h incubation at room temperature with an Alexa Fluor 488-conjugated secondary antibody (1:500, Invitrogen, USA), and nuclei were counterstained with DAPI for 10 min. Coverslips were mounted with anti-fade mounting medium (P0126, Beyotime, China). Images were acquired with a digital slide scanner (VS120-S6-W, Japan) and analyzed accordingly.

### Fluorometric assay of caspase-1 and caspase-3 activities

Caspase-1 and Caspase-3 fluorescent probes (developed by Zhejiang University) were used to detect cell pyroptosis and apoptosis, respectively. Cells in the logarithmic growth phase were seeded into 24-well plates and for grouped as described above. After culturing for 48 h, Caspase-1 and Caspase-3 probes were diluted to a 1:1000 ratio with fresh medium. Following a 1 h incubation, the cells were stained with Hoechst 33,342 (C1028, Beyotime, China) for 10 min. The cells were observed and imaged using a fluorescence inverted microscope live cell imaging system, and the photographed areas were then quantified using Image-J software.

### Western blot

Proteins were extracted with RIPA buffer and quantified by BCA assay. Samples (30 μg) were separated by SDS-PAGE, transferred to PVDF membranes, and incubated with primary antibodies against NQO1 (1:1000, 62262 T, CST, USA), TNF-α (1:800, 11948 T, CST, USA), NF-κB (1:5000, 80979-1-RR, Proteintech, China), and p-NF-κB (1:2000, 82335-1-RR, Proteintech, China). HRP-conjugated secondary antibody (1:5000) and ECL reagent (P0018S, Beyotime, China) were used for detection. β-Actin served as the loading control.

### Quantitative reverse-transcription PCR (qRT-PCR)

Total RNA was extracted using the RNAeasy™ Animal RNA Extraction Kit (Beyotime, R0026) in strict accordance with the manufacturer’s protocol. RNA concentration was subsequently determined using a Nanodrop One spectrophotometer (USA). Reverse transcription of RNA into cDNA was carried out with the SeqHunt® kit (Seq-Hunt Biotechnology, 25C03) following the provided instructions. Quantitative real-time PCR (qPCR) was then performed to assess target mRNA expression using the 2 × Universal SYBR Master Mix (Seq-Hunt Biotechnology, 25A09) under standard reaction conditions. Primer sequences used in the qPCR assays are as follows: SMOX, forward, 5ʹ-CGGATGACCCTCTCAGTCG-3ʹ and reverse, 5ʹ-GCGTGTCCAAGTTTCACACT-3ʹ. TNF-α, forward, 5ʹ-GCTGCACTTTGGAGTGATCG-3ʹ and reverse, 5ʹ-GCTTGAGGGTTTGCTACAACA-3ʹ. EDIL3, forward, 5ʹ-AGCATACCGAGGAGATACATT-3ʹ and reverse, 5ʹ-CAAGGCTCAACTTCGCATTCA-3ʹ. FGF2, forward, 5ʹ-CCCAGAAAACCCGAGCGA-3ʹ and reverse, 5ʹ-CGCGGCGTCACATCTTCTA-3ʹ. β-actin, forward, 5ʹ-CTCCATCCTGGCCTCGCTGT-3ʹ and reverse, 5ʹ-GCTGCTACCTTCACCGTTCC-3ʹ.

### Single-cell sequencing

Publicly available single-cell RNA sequencing (scRNA-seq) data of diabetic foot ulcers (DFU) were retrieved from the GEO database (GSE165816). The analysis focused on two sample groups: Non-healing DFU patients and healthy non-diabetic controls. Processed in R with Seurat (v4.3.0): low-quality cells (< 200 genes, > 5% mitochondrial content) and doublets were filtered out. Data were normalized, and variable genes (top 2000) were selected. Batch effects were corrected using Harmony, followed by PCA (30 PCs) and Louvain clustering (resolution 0.5) with UMAP visualization. Endothelial cells were subsetted via canonical markers (PECAM1, VWF) and annotated with SingleR. Differential expression analysis in this subset (Seurat’s FindMarkers, Wilcoxon test) identified DEGs with |log2FC|> 1 and adj. *P* < 0.05.

### Statistical analysis

Data were presented as mean ± SEM and analyzed using GraphPad Prism 9 software. Comparisons between two groups were made using t-tests or non-parametric tests, as appropriate. For comparisons involving multiple groups, one-way ANOVA followed by post-hoc multiple comparison tests were applied. *P* < 0.05 was considered significant.

## Results

### HET-B improves perfusion and attenuates inflammation in a murine model of diabetic limb ischemia

**Fig. 1:**
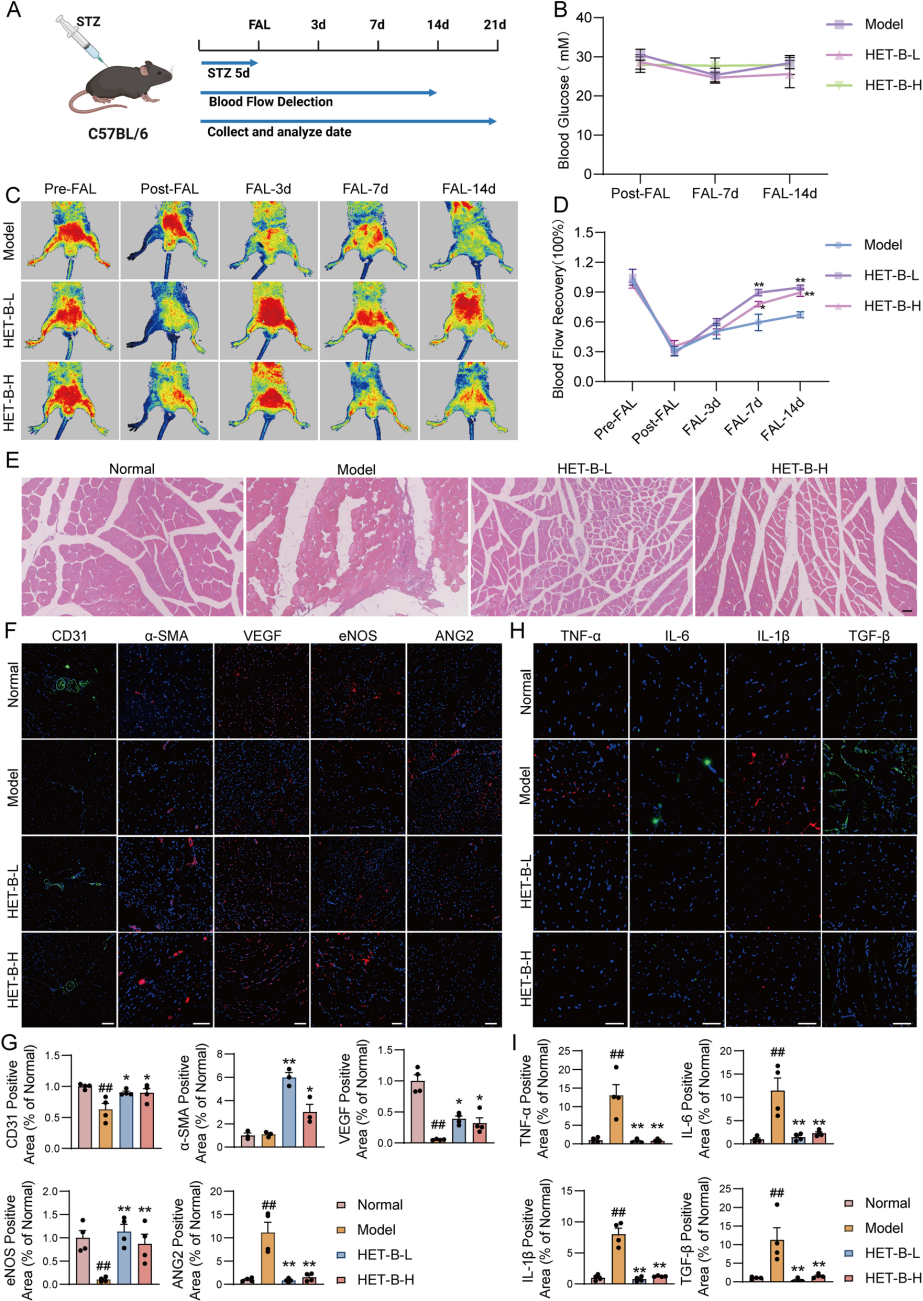
HET-B improves perfusion and attenuates inflammation in DLLI mice. **A** Schematic diagram of the animal experimental design. **B** Random blood glucose levels in mice, no significant differences were observed between HET-B-treated groups and the model group. **C** Representative laser Doppler perfusion images showing hindlimb blood flow recovery at indicated time points post-surgery. **D** Statistical chart of the perfusion ratio (ischemic/non-ischemic limb), reflecting tissue perfusion recovery. **E** Representative H&E staining images of gastrocnemius muscle sections, showing tissue morphology and inflammatory cell infiltration. **F** Immunofluorescence staining of CD31, α-SMA, VEGF, eNOS, and ANG2 in gastrocnemius muscle. **G** Quantitative analysis of CD31, α-SMA, VEGF, eNOS, and ANG2 positive area, respectively. **H** Immunofluorescence staining of TNF-α, IL-6, IL-1β, and TGF-β in gastrocnemius muscle. **I** Quantitative analysis of TNF-α, IL-6, IL-1β, and TGF-β positive area, respectively. n = 4 per group. Scale bar = 50 μm. ^##^*P* < 0.01 *versus* Normal; ^*^P < 0.05, ^**^P < 0.01 versus Model

To evaluate the therapeutic potential of HET-B, we established a murine model of DLLI (Fig. 1A). Notably, HET-B treatment did not significantly alter random blood glucose levels compared to the diabetic model group throughout the experimental period, suggesting its effects are independent of glycemic control (Fig. 1B). HET-B treatment (3 or 6 mg/kg) significantly enhanced blood flow recovery from day 7 post-surgery onwards, as assessed by laser Doppler imaging, compared to the Model group (Fig. 1C, D).

Histological analysis by H&E staining revealed that DLLI mice exhibited marked muscle fiber disorganization, necrosis, and inflammatory cell infiltration, which were significantly ameliorated by HET-B treatment (Fig. 1E). Immunofluorescence analysis of gastrocnemius muscle sections showed that HET-B-treated mice had increased expression of CD31, α-SMA, VEGF, and eNOS, along with decreased Angiopoietin-2 (ANG2) expression (Fig. 1F, G), indicating potent promotion of mature and functional angiogenesis. Concurrently, HET-B administration substantially reduced the expression of key pro-inflammatory cytokines, including TNF-α, IL-6, and IL-1β, as well as the pleiotropic cytokine TGF-β, in the ischemic muscle tissue (Fig. 1H, I).

### HET-B restores high glucose-impaired endothelial cell function in vitro

**Fig. 2:**
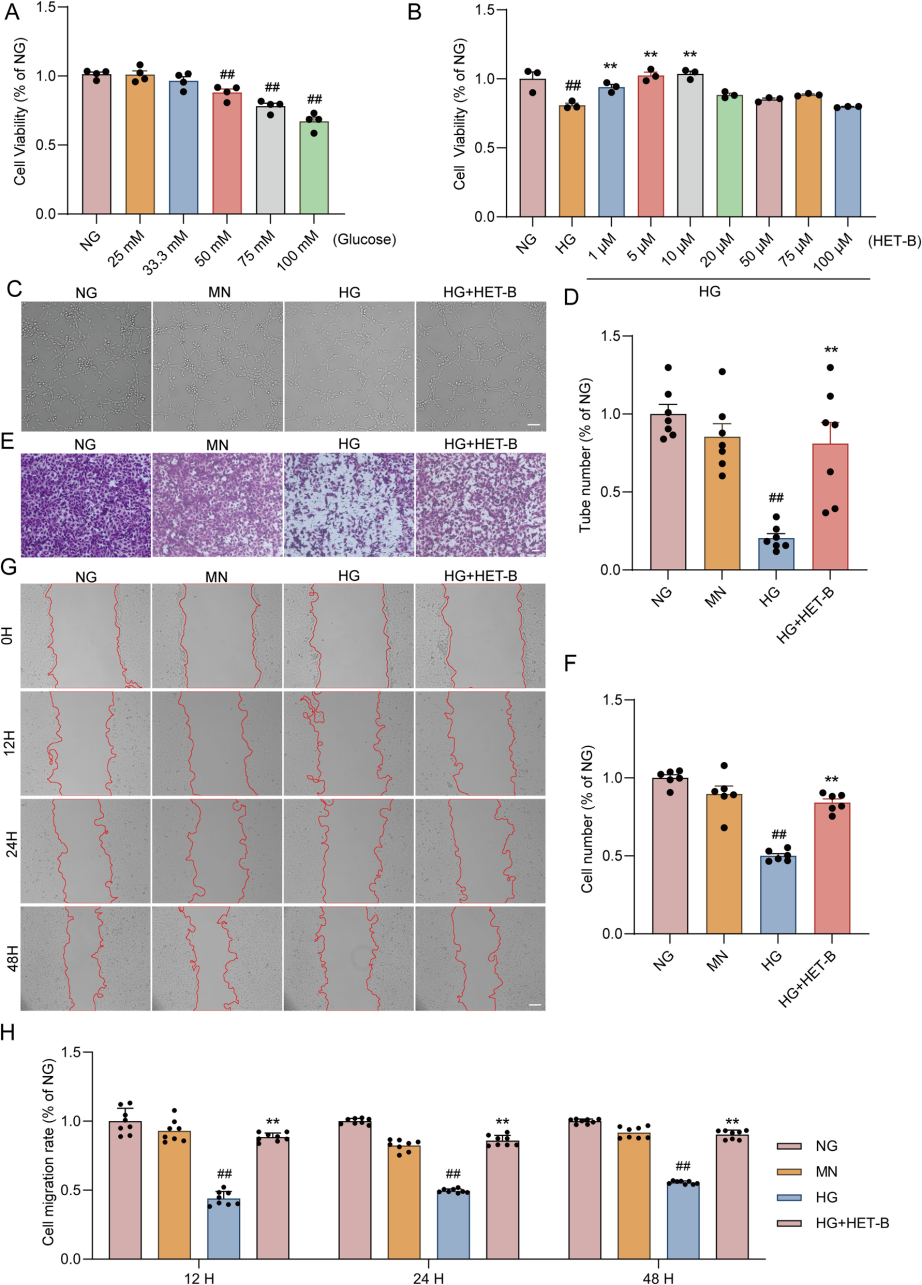
HET-B restores endothelial cell function under high glucose conditions. **A** Cell viability under different glucose concentrations. **B** Cell viability with different HET-B concentrations. **C** Representative images of tube formation assay. **D** Quantitative analysis of tube formation. **E** Transwell migration assay. **F** Quantitative analysis of cell migration. **G** Wound healing assay. **H** Quantitative analysis of wound closure rate. Scale bar = 50 μm. ^##^*P* < 0.01 versus NG; ^**^P < 0.01 versus HG

To determine whether HET-B ameliorates high glucose-induced angiogenesis impairment, we established an in vitro model using HUVECs cultured under HG conditions. The CCK-8 assay was used to determine the optimal HG concentration for modeling (50 mM) and the effective concentration of HET-B (10 μM) (Fig. 2A, B). Cells were treated with HG (50 mM) or maintained in an iso-osmotic control medium containing 5.6 mM glucose plus 44.5 mM mannitol for 48 h. The MN group served as an osmotic control to account for potential hyperosmotic stress induced by HG. Tube formation assays revealed that HG significantly impaired angiogenic capacity, whereas HET-B treatment markedly restored this functionality. No significant difference was observed between the MN and HG groups (Fig. 2C, D). In Transwell migration assays, hyperosmolarity showed no notable effect on cell migration; however, HG exposure significantly suppressed migration, an effect that was reversed by HET-B administration (Fig. 2E, F). Wound healing assays demonstrated that the HG group exhibited a significantly reduced migration rate over time compared to the normal glucose (NG) group, and this impairment was significantly rescued by HET-B treatment (Fig. 2G, H). The lack of effect in the MN group confirms that the impairments are due to high glucose metabolism rather than hyperosmolarity. Collectively, these results indicate that HET-B enhances proliferation, migration, and tube-forming ability of HUVECs under HG conditions in vitro.

### HET-B activates the Nrf2 antioxidant pathway and mitigates oxidative stress in HUVECs

**Fig. 3:**
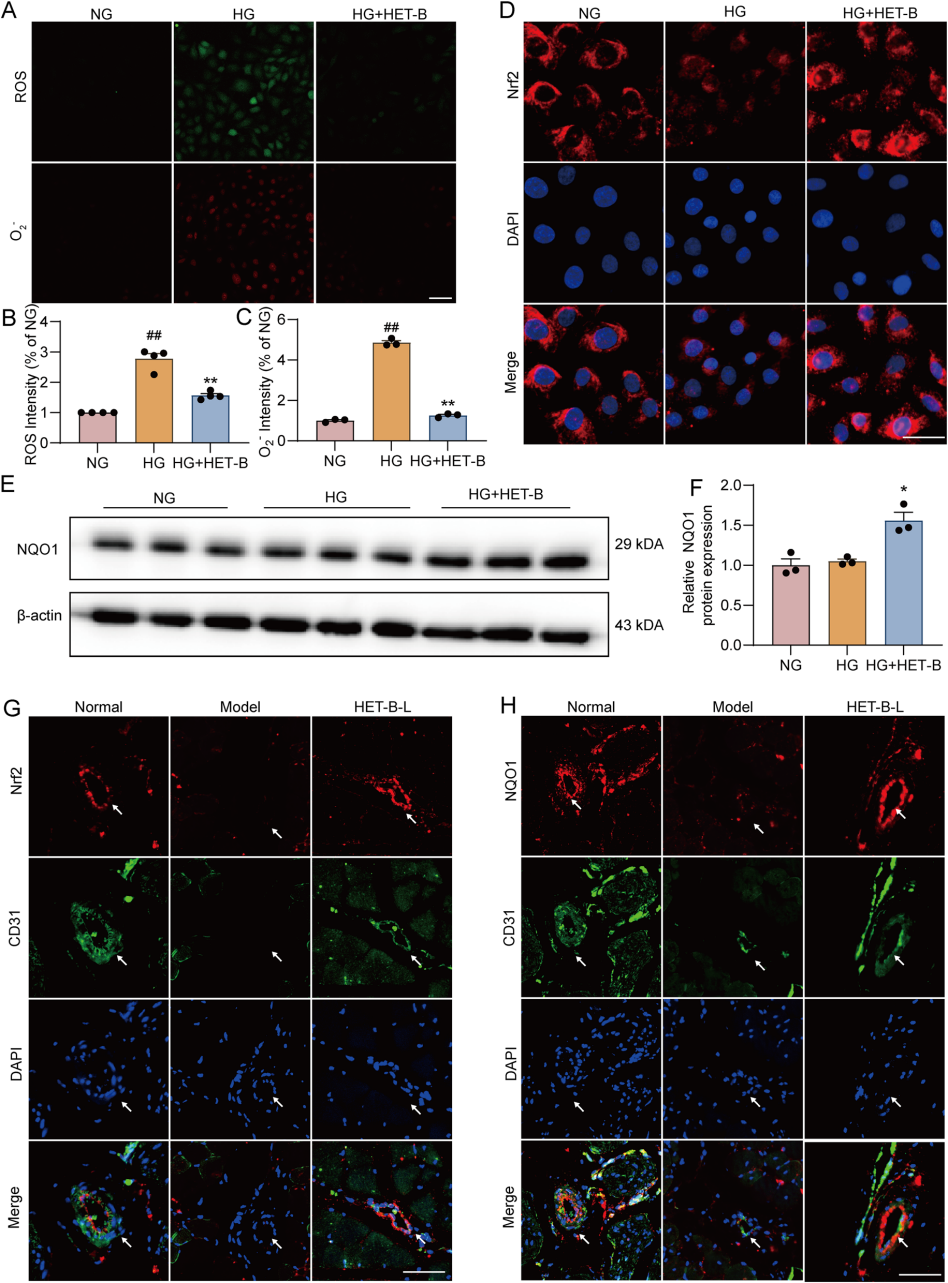
HET-B activates Nrf2 pathway and alleviates oxidative stress. **A** Representative fluorescence images of ROS and O₂⁻. **B** Statistical chart of ROS mean fluorescence intensity. **C** Statistical chart of O₂⁻ mean fluorescence intensity. **D** Representative immunofluorescence images showing Nrf2 nuclear translocation in HUVECs. **E** Western blot analysis of NQO1. **F** Quantitative analysis of NQO1 protein expression. **G** Representative immunofluorescence co-staining images of Nrf2 (red) and CD31 (green) in gastrocnemius muscle. **H** Representative immunofluorescence co-staining images of NQO1 (red) and CD31 (green) in gastrocnemius muscle. Scale bar = 50 μm. ^#^*P* < 0.05, ^##^*P* < 0.01 versus NG or Normal; ^*^P < 0.05, ^**^P < 0.01 versus HG or Model

Given the central role of oxidative stress in diabetic endothelial dysfunction, we investigated the antioxidant mechanism of HET-B. HG stimulation markedly increased the accumulation of intracellular ROS and superoxide anion (O₂⁻) in HUVECs, whereas HET-B treatment effectively suppressed the levels of both ROS (Fig. 3A–C). Mechanistically, immunofluorescence staining revealed that HET-B promoted the nuclear translocation of Nrf2 in HUVECs (Fig. 3D). Consistent with Nrf2 activation, Western blot analysis demonstrated that HET-B up-regulated the expression of the downstream antioxidant enzyme NQO1 (Fig. 3E, F). To further validate these findings in vivo, we assessed Nrf2 and NQO1 expression in the ischemic muscle tissues. Immunofluorescence co-staining with the endothelial marker CD31 confirmed that Nrf2 and NQO1 were expressed in endothelial cells. Their expression was decreased in DLLI mice compared to controls, and this reduction was reversed by HET-B treatment (Fig. 3G, H). These findings collectively indicate that HET-B alleviates oxidative stress by activating the Nrf2/NQO1 signaling pathway in both cellular and animal models of diabetic vascular injury.

### HET-B suppresses high glucose-induced inflammation, pyroptosis and apoptosis in HUVECs

**Fig. 4:**
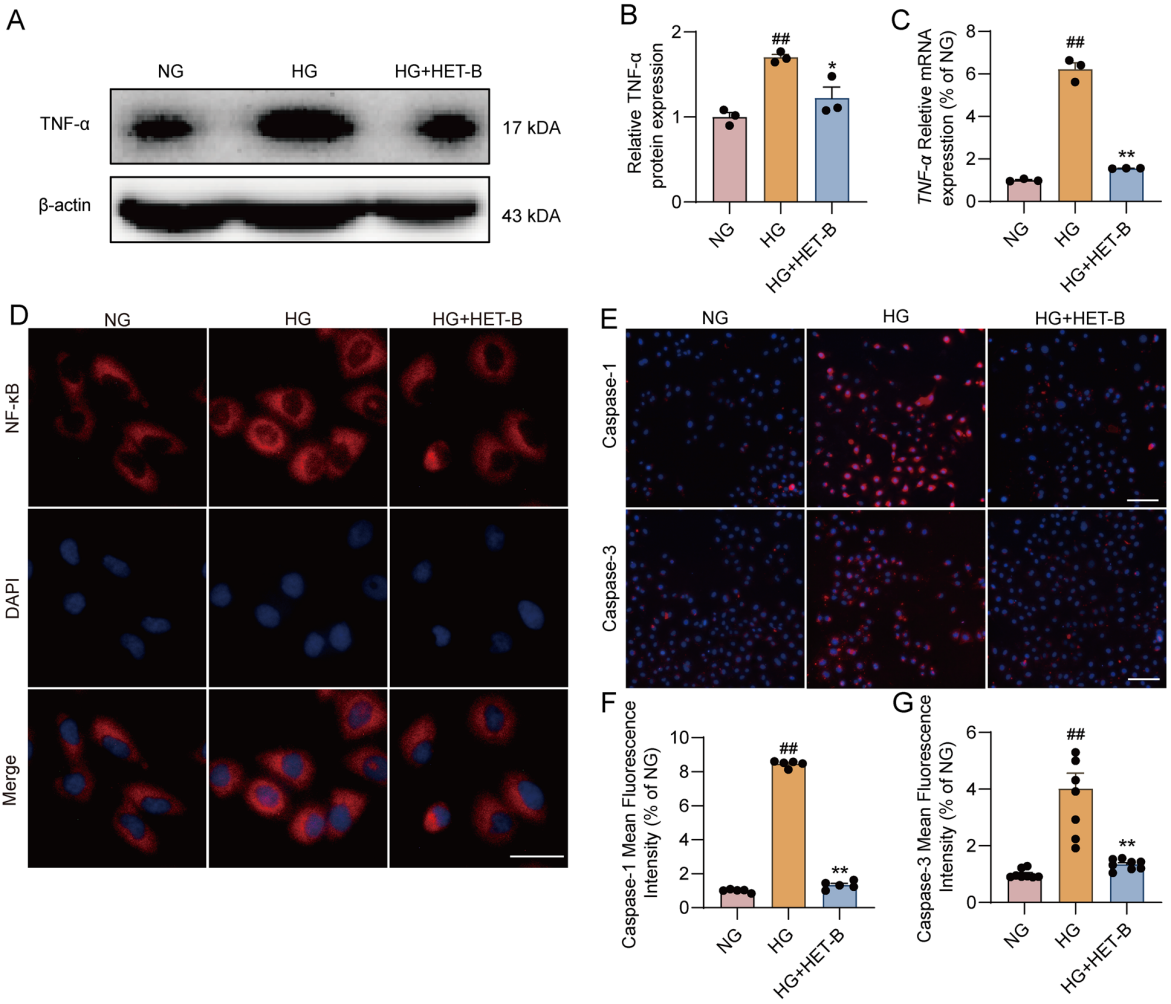
HET-B suppresses inflammation, pyroptosis and apoptosis in HUVECs. **A** Western blot analysis of TNF-α. **B** Quantitative analysis of TNF-α protein expression. **C** TNF-α mRNA expression levels. **D** Representative immunofluorescence images showing NF-κB nuclear translocation in HUVECs. **E** Caspase-1 and Caspase-3 fluorescence staining. **F** Statistical chart of Caspase-1 mean fluorescence intensity. **G** Statistical chart of Caspase-3 mean fluorescence intensity. Scale bar = 50 μm. ^##^*P* < 0.01 versus NG; ^*^P < 0.05, ^**^P < 0.01 versus HG

HG-induced oxidative stress is known to trigger inflammatory and apoptotic responses in endothelial cells. We found that HET-B significantly suppressed the HG-induced upregulation of the pro-inflammatory cytokine TNF-α, both at the protein level (Fig. 4A, B) and the mRNA level (Fig. 4C). To further investigate the underlying inflammatory signaling, we assessed NF-κB p65 nuclear translocation by immunofluorescence. HG stimulation markedly promoted p65 nuclear translocation, indicating NF-κB pathway activation, whereas HET-B treatment effectively reversed this effect (Fig. 4D). Furthermore, the evaluation of cell death markers revealed that HG stimulation markedly increased the activity of Caspase-1 (indicating pyroptosis) and Caspase-3 (indicating apoptosis), effects that were markedly reversed by HET-B treatment (Fig. 4E–G). Taken together, these results demonstrate that HET-B effectively protects HUVECs from HG-induced inflammatory activation, pyroptosis, and apoptosis.

### Identification of SMOX as a key metabolic mediator and its suppression by HET-B.

**Fig. 5:**
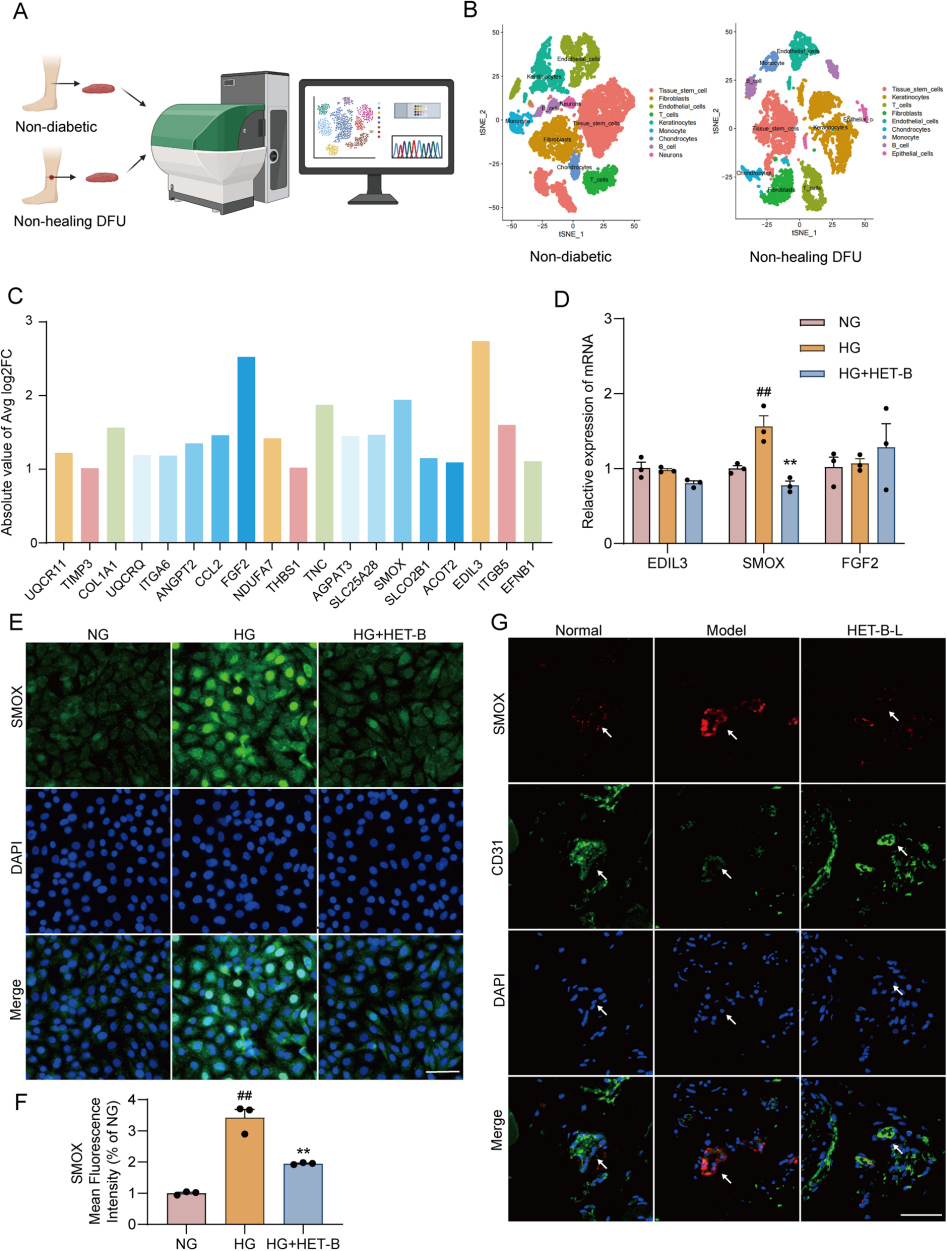
SMOX identification as a metabolic mediator of HET-B. **A** Workflow of bioinformatic analysis. **B** UMAP visualization of endothelial cell clusters. **C** Volcano plot of differentially expressed genes. **D** mRNA expression levels of SMOX, EDIL3, and FGF2 determined by qRT-PCR. **E** SMOX immunofluorescence in HUVECs. **F** Quantitative analysis of SMOX mean fluorescence intensity in cells. **G** Representative immunofluorescence co-staining images of SMOX (red) and CD31 (green) in gastrocnemius muscle. Scale bar = 50 μm. ^##^*P* < 0.01 versus NG; ^*^P < 0.01 versus HG

To uncover upstream regulators, we analyzed a public single-cell RNA sequencing dataset (GSE165816) from diabetic foot ulcers. Bioinformatic analysis identified SMOX (Spermine Oxidase) as one of the top differentially expressed gene at the intersection of metabolic and angiogenic pathways in endothelial cells from non-healing diabetic wounds (Fig. 5C). Validation experiments confirmed that HG stimulation significantly elevated SMOX mRNA and protein expression in HUVECs, and promoted its nuclear localization. HET-B treatment effectively suppressed this HG-induced overexpression and aberrant nuclear translocation of SMOX (Fig. 5D–F). Consistent with the in vitro findings, immunofluorescence co-staining with the endothelial marker CD31 confirmed that SMOX expression was markedly increased in endothelial cells of ischemic muscle tissues from Model mice, and this upregulation was significantly reversed by HET-B treatment (Fig. 5G). These results position SMOX as a critical metabolic mediator of diabetic endothelial injury and a key mediator of the protective effects of HET-B.

### SMOX inhibition mediates the protective effects of HET-B

**Fig. 6:**
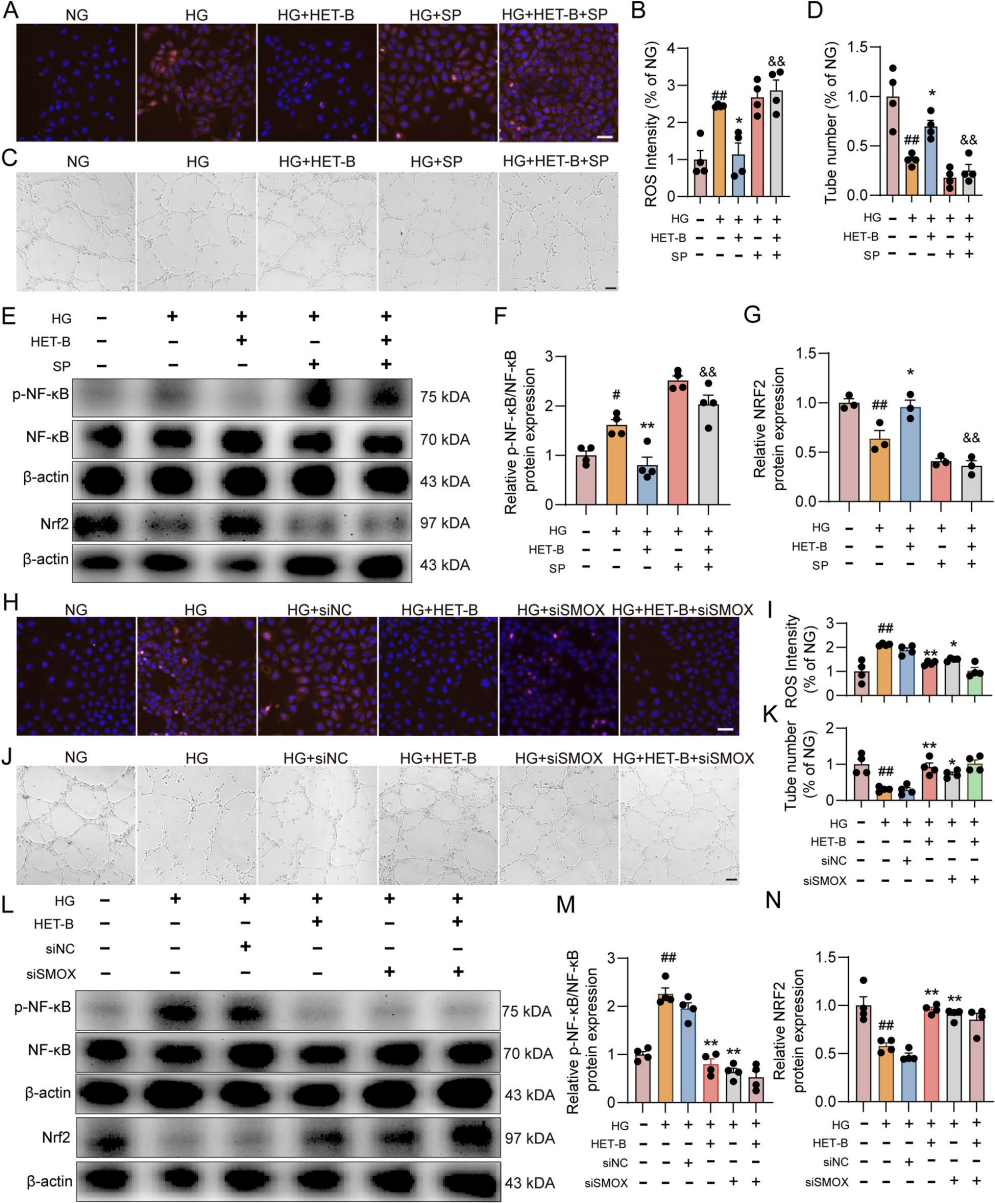
SMOX inhibition mediates the protective effects of HET-B. **A** Representative fluorescence images of ROS in HUVECs. **B** Quantitative analysis of ROS mean fluorescence intensity. **C** Representative images of tube formation. **D** Quantitative analysis of tube formation. **E** Western blot analysis of Nrf2 and p-NF-κB protein expression. **F** Densitometric quantification of Nrf2 protein levels. **G** Densitometric quantification of p-NF-κB protein levels. **H** Representative fluorescence images of ROS in HUVECs. **I** Quantitative analysis of ROS mean fluorescence intensity. **J** Representative images of tube formation. **K** Quantitative analysis of tube formation. **L** Western blot analysis of Nrf2 and p-NF-κB protein expression. **M** Densitometric quantification of Nrf2 protein levels. **N** Densitometric quantification of p-NF-κB protein levels. Scale bar = 50 μm. ^#^*P* < 0.05, ^##^*P* < 0.01 versus NG; ^*^P < 0.05, ^**^P < 0.01 versus HG; ^&^*P* < 0.05, ^&&^*P* < 0.01 versus HG + HET-B

To determine whether the protective effects of HET-B are mediated through SMOX inhibition, rescue experiments were performed using the SMOX activator spermine (SP) and SMOX-targeting siRNA. Activation of SMOX by SP largely abrogated the protective effects of HET-B. In the presence of SP, the HET-B-induced reduction in ROS production (Fig. 6A, B) and improvement in tube formation (Fig. 6C, D) were both reversed. Consistently, Western blot analysis revealed that SP treatment abolished HET-B-induced upregulation of Nrf2 and downregulation of p-NF-κB p65 (Fig. 6E–G). In contrast, knockdown of SMOX using specific siRNA (siSMOX) mimicked the protective effects of HET-B. Under HG conditions, siSMOX significantly reduced ROS production (Fig. 6H, I), enhanced tube formation (Fig. 6J, K), increased Nrf2 expression, and decreased p-NF-κB p65 levels (Fig. 6L–N). Notably, the addition of HET-B did not further enhance these effects in siSMOX-treated cells, suggesting that HET-B and SMOX knockdown act through a shared pathway. Collectively, these findings indicate that SMOX inhibition is both necessary and sufficient for the protective effects of HET-B on endothelial function, Nrf2 activation, and NF-κB suppression.

## Discussion

Our findings demonstrate that HET-B confers significant protection against DLLI independently of glycemic control. In vivo, HET-B robustly enhanced limb perfusion, promoted angiogenesis, and suppressed the expression of local inflammatory cytokines in DLLI mice, thereby establishing a direct, glucose-independent vasculoprotective function. To elucidate the underlying cellular mechanisms, we employed a high glucose-induced endothelial injury model. HET-B effectively restored the impaired migration, proliferation, and tube-forming capacity of endothelial cells, which are functional endpoints critical for vascular repair under ischemic conditions.

The core pathological axis of DLLI involves a vicious cycle linking oxidative stress and chronic inflammation [20, 21]. In endothelial cells, high glucose induces mitochondrial dysfunction and a consequent overproduction of ROS and O₂⁻, creating a state of pronounced oxidative stress [22]. HET-B treatment potently attenuated this oxidative burden. The transcription factor Nrf2 is a master regulator of the cellular antioxidant response, and its activation is crucial for counteracting hyperglycemia-induced damage [23, 24]. We found that HET-B promoted the nuclear translocation of Nrf2 and upregulated its key downstream effector, NQO1, in both cellular and animal models. The activation of the Nrf2/NQO1 pathway represents a primary mechanism through which HET-B restores redox homeostasis. Concurrently, oxidative stress is a potent activator of pro-inflammatory signaling, notably the NF-κB pathway, which drives the expression of cytokines such as TNF-α [11, 25]. Importantly, Nrf2 activation itself can impede NF-κB signaling by alleviating the cellular redox burden, thereby breaking this vicious cycle [26, 27]. Consistent with this interplay, our data show that HG stimulation promoted NF-κB p65 nuclear translocation and elevated TNF-α levels and activated Caspase-1 and Caspase-3, mediators of inflammatory cell death and apoptosis, respectively. HET-B treatment effectively suppressed these events. Thus, the antioxidant action of HET-B via Nrf2 is intrinsically linked to its observed anti-inflammatory and anti-apoptotic effects, providing a coordinated defense against multifaceted endothelial injury. We also observed that HET-B significantly reduced TGF-β expression in ischemic tissue. TGF-β exerts complex, context-dependent effects on angiogenesis through the SMAD signaling pathway: activation of ALK5/SMAD2/3 signaling generally promotes fibrosis and inhibits angiogenesis, whereas ALK1/SMAD1/5/8 signaling can be pro-angiogenic [28–30]. The net outcome depends on receptor usage and crosstalk with other pathways such as VEGF and NF-κB [31, 32]. The reduction of TGF-β by HET-B may therefore contribute to tilting the balance toward a pro-angiogenic milieu, complementing its direct antioxidant and anti-inflammatory actions.

To identify the upstream regulator initiating this protective cascade, we performed bioinformatic analysis of clinical single-cell RNA sequencing data from diabetic foot ulcers. This analysis identified SMOX as a critically dysregulated gene at the intersection of metabolic and vascular pathways in diabetic endothelium. SMOX is a metabolic enzyme that generates H₂O₂ during polyamine catabolism [33]. H₂O₂ is an important reactive ROS, and its abnormal accumulation disrupts the cellular redox balance [34, 35]. Critically, emerging evidence indicates that SMOX is a key upstream regulator of the Nrf2 pathway, inhibition of SMOX can activate Nrf2 and its downstream target heme oxygenase-1 (HO-1), thereby mitigating oxidative stress and cellular damage in various disease models [36–38]. Our experimental validation confirmed that HG induces SMOX overexpression at both the mRNA and protein levels, and that HET-B treatment reverses this process both in vitro and in vivo. Consistent with this, SMOX knockdown mimicked the protective effects of HET-B, whereas activation of SMOX with its substrate spermine abrogated these protective effects, reinforcing the causal role of SMOX inhibition in the therapeutic action of HET-B. Therefore, we propose that SMOX serves as a key upstream metabolic mediator of HET-B. By inhibiting SMOX, HET-B reduces the enzymatic production of pro-oxidant molecules, thereby alleviating the suppression on the Nrf2 pathway. The subsequent activation of Nrf2 orchestrates the downstream antioxidant defense and contributes to the dampening of inflammatory signaling. This integrated mechanism, whereby HET-B inhibits SMOX to activate the Nrf2 pathway and mitigate inflammation, provides a coherent explanation for its multi-target protective efficacy against the complex pathology of DLLI.

From a TCM perspective, *Pseudostellaria heterophylla* (Tai Zi Shen) is well-documented for its actions in replenishing *Qi* and nourishing *Yin*. *Qi* deficiency is often associated with impaired energy metabolism and compromised tissue repair, while *Yin* deficiency frequently manifests as hyperactive oxidative stress and inflammatory responses, collectively termed “pathogenic heat” or “toxin” in TCM theory [14, 39]. In this study, HET-B, a major cyclopeptide derived from this herb, promoted angiogenesis and mitigated oxidative stress and inflammation in diabetic ischemic tissues. These effects parallel the traditional functions of replenishing *Qi* to support tissue regeneration and nourishing *Yin* to clear “oxidative toxins”. Thus, the molecular mechanism involving SMOX inhibition and Nrf2 activation provides a modern scientific correlate to the classical TCM understanding of *Qi* and *Yin* reinforcement, bridging traditional ethnopharmacological knowledge with contemporary mechanistic insights.

Several limitations of this study should be acknowledged. First, while this study delineates the SMOX-Nrf2 axis as central to HET-B's action, future work should explore the precise molecular interaction between HET-B and the SMOX protein. Second, the causal role of SMOX in this protective mechanism was primarily demonstrated through in vitro knockdown and pharmacological activation experiments; in vivo validation using SMOX knockout animals would provide more definitive evidence. Third, the sample size of animals used in this study, although sufficient to detect statistically significant differences, is relatively modest; future studies with larger animal cohorts would further strengthen the statistical power and reliability of the findings.

In summary, our work suggests that HET-B alleviates DLLI by inhibiting SMOX, thereby activating the Nrf2 antioxidant pathway and suppressing inflammatory and apoptotic processes. From a translational perspective, it would be of great interest to investigate whether plasma or tissue SMOX levels correlate with DLLI severity or could serve as a predictive biomarker for therapeutic response to HET-B. Such findings would not only facilitate patient stratification but also provide a clinically accessible tool for monitoring treatment efficacy. Furthermore, evaluating the efficacy of HET-B in disease models with comorbidities and assessing its long-term safety profile will be essential steps toward therapeutic translation.

## Conclusions

This study demonstrates that HET-B ameliorates DLLI by inhibiting SMOX, thereby activating the Nrf2 antioxidant pathway and suppressing inflammation. These findings suggest that targeting the SMOX-Nrf2 axis may represent a potential therapeutic strategy for DLLI.

## Supplementary Information

Supplementary material 1.

## Data Availability

The data that support the findings of this study are available on request from the corresponding author.

## Abbreviations

ANG2: Angiopoietin-2
DLLI: Diabetic lower limb ischemia
FAL: Femoral artery ligation
HET‑B: Heterophyllin B
HG: High glucose
HUVECs: Human umbilical vein endothelial cells
MN: Mannitol
NG: Normal glucose
NQO1: NAD(P)H quinone oxidoreductase 1
Nrf2: Nuclear factor erythroid 2‑related factor 2
SMAD: Mothers against decapentaplegic homolog
SMOX: Spermine oxidase
SP: Spermine
STZ: Streptozotocin
TCM: Traditional Chinese Medicine
