# Supplementary material for: Heterophyllin B ameliorates diabetic lower limb ischemia by inhibiting SMOX to activate the Nrf2 antioxidant pathway
Source: Chin Med. 2026 Jun 10;21:167. doi: 10.1186/s13020-026-01440-x (PMC13255290; doi:10.1186/s13020-026-01440-x)
Supplement: Supplementary file 1 — Supplementary material 1. [file 13020_2026_1440_MOESM1_ESM.pdf]

1.FIG3 E

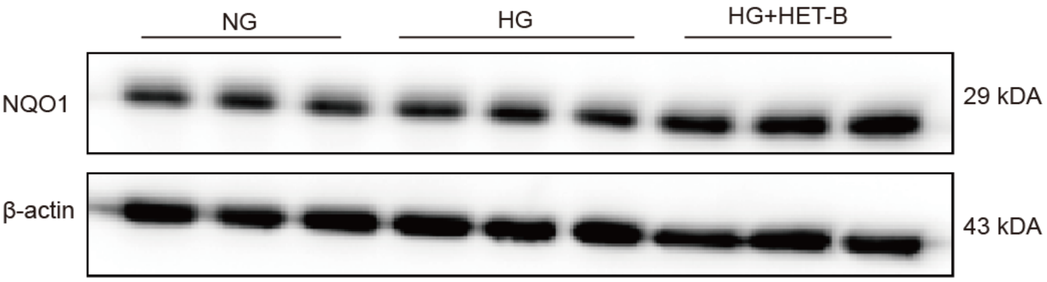

➢ NQO1

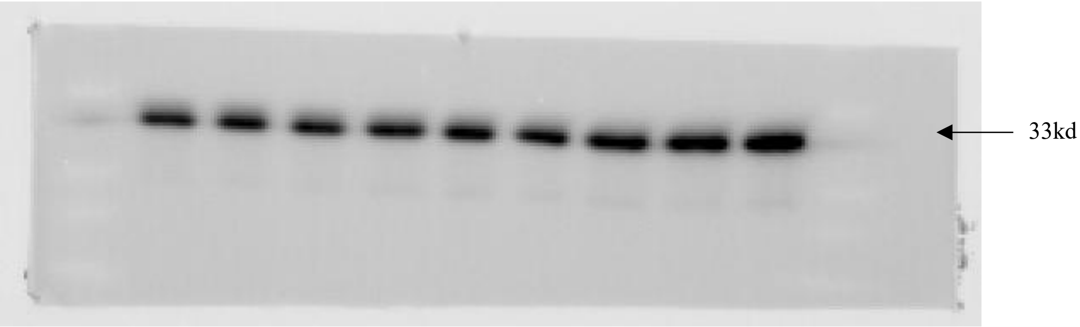

33kd

➢ β-actin

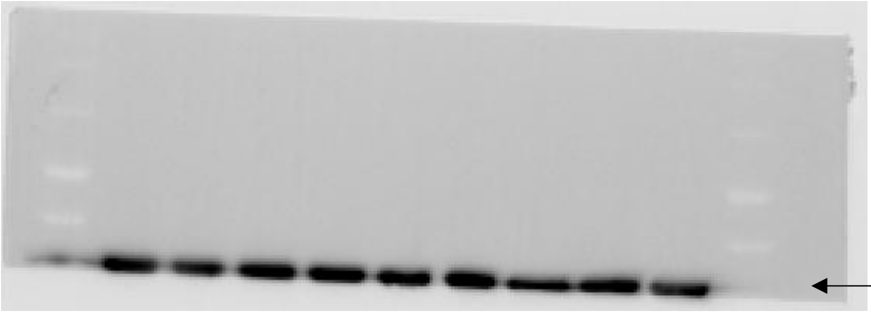

43kd

➢ NQO1-β-actin

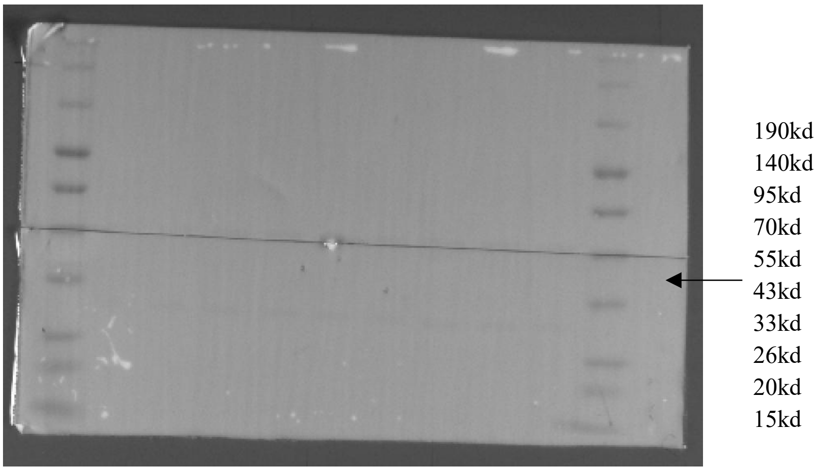

190kd
140kd
95kd
70kd
55kd
43kd
33kd
26kd
20kd
15kd

2.FIG4 A

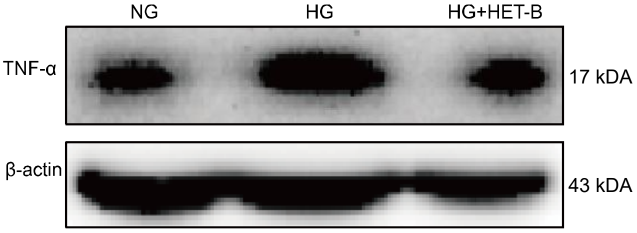

- TNF-α

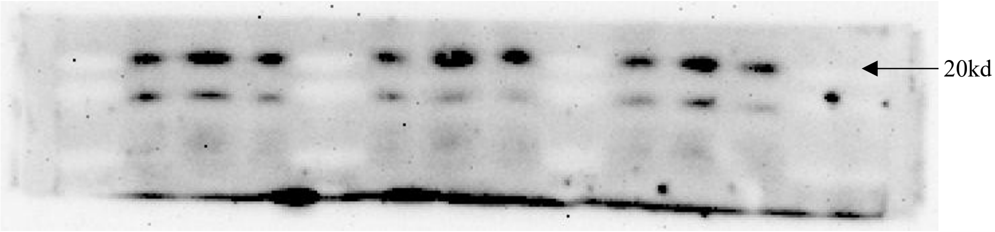

- β-actin

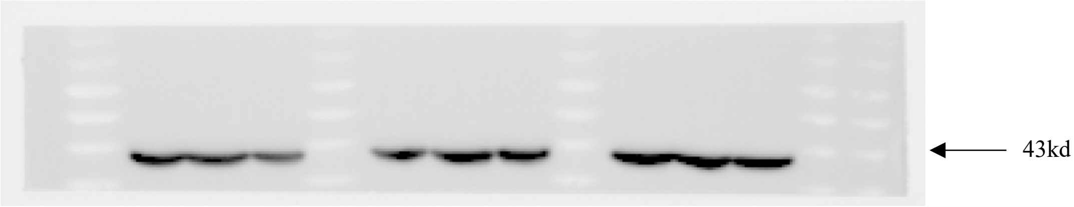

- TNF-α-β-actin

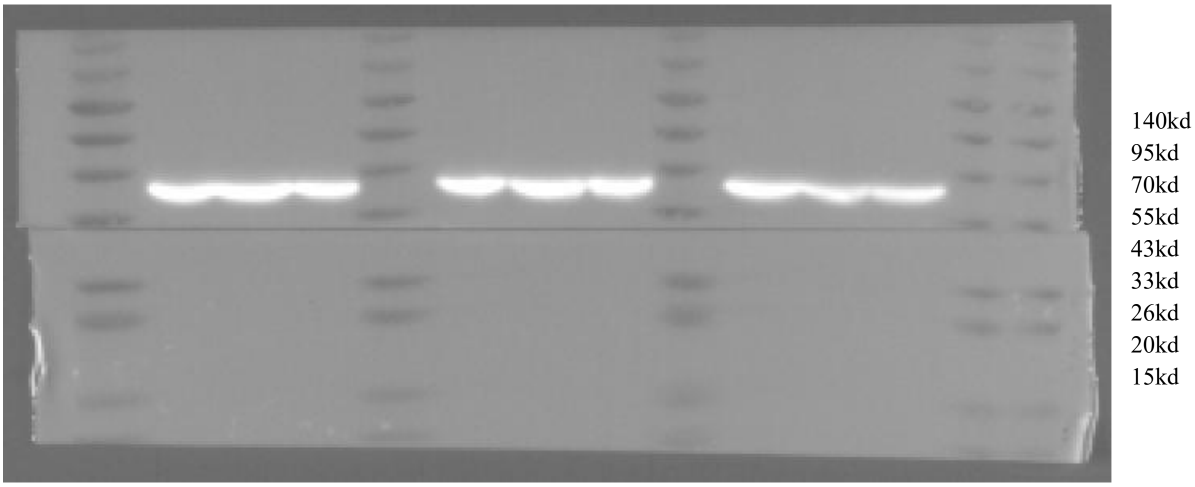

3.FIG6 E

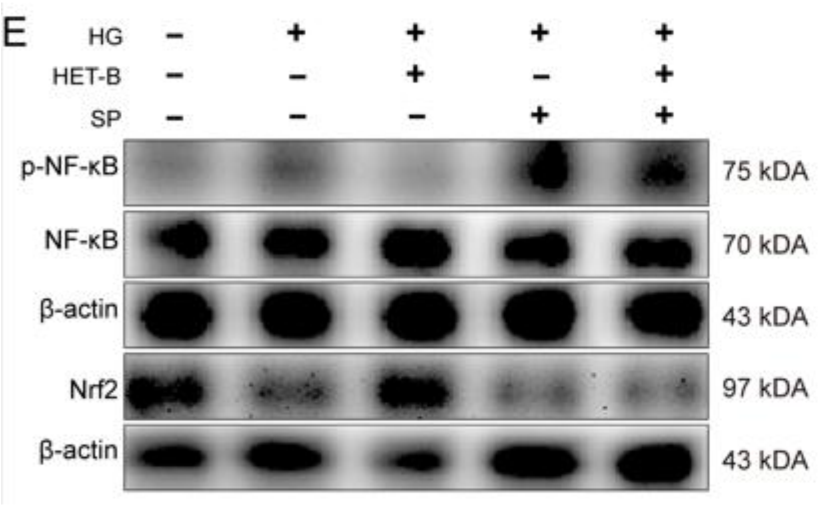

- ① p-NF-κB

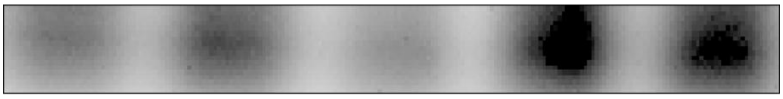

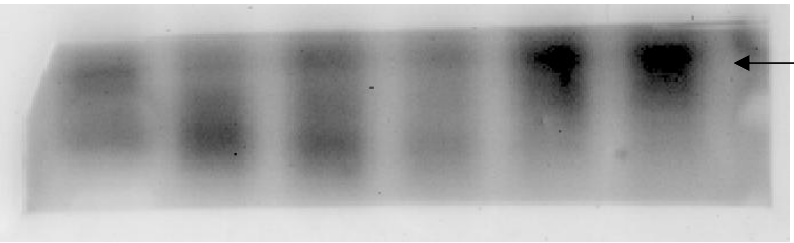

75kd

- ① NF-κB

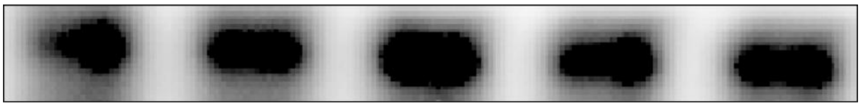

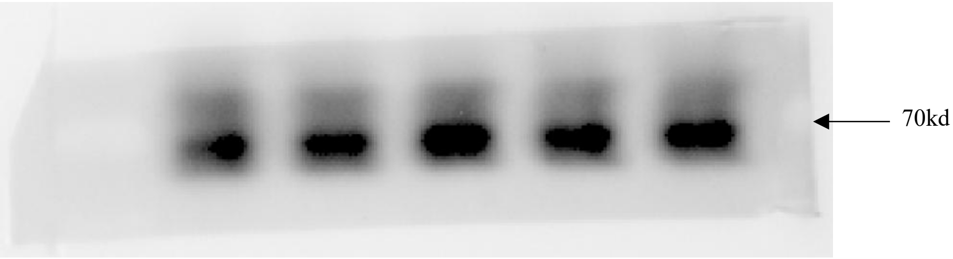

70kd

- ① β-actin (NF-κB)

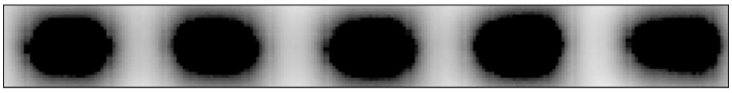

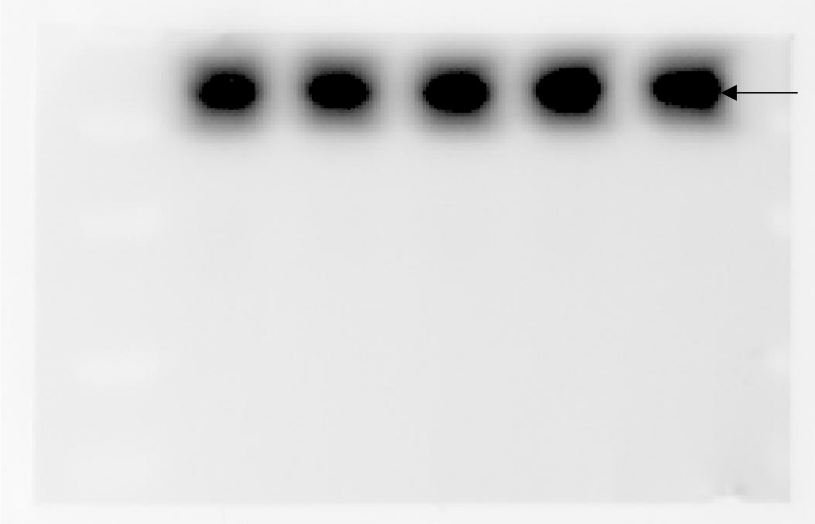

➢ ① white light (NF-κB)

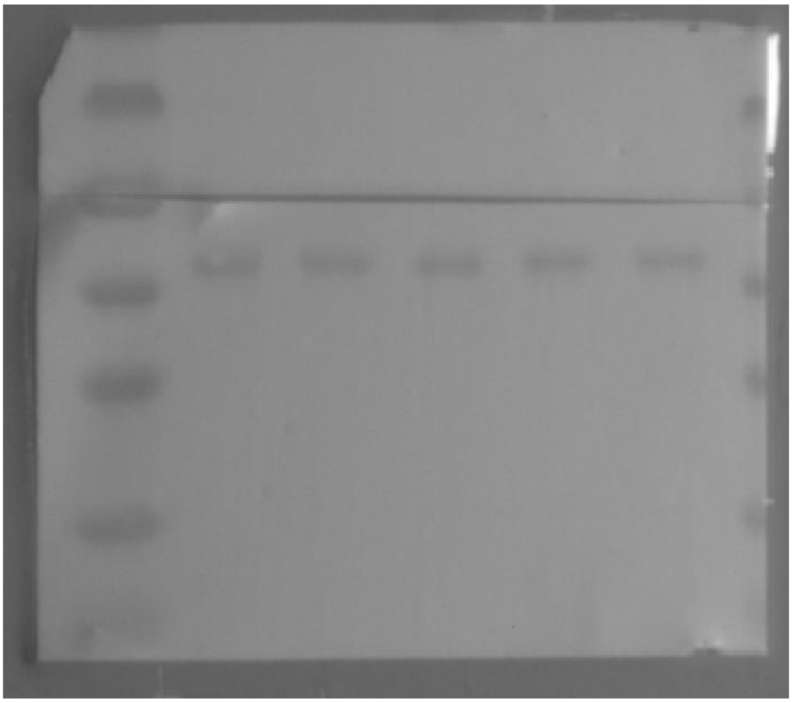

➢ ② p-NF-κB

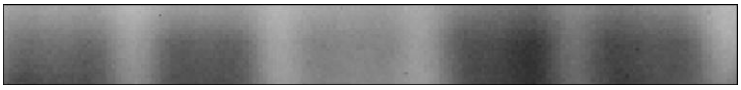

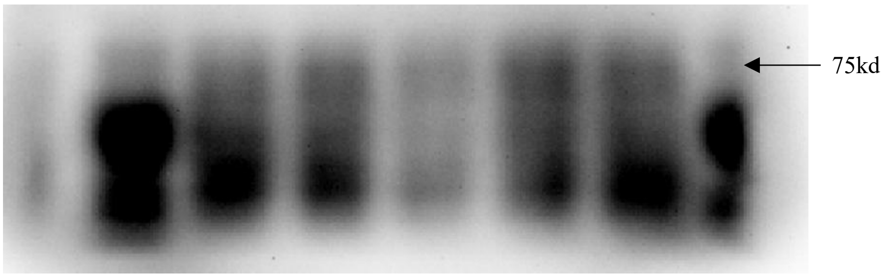

- ② NF-κB

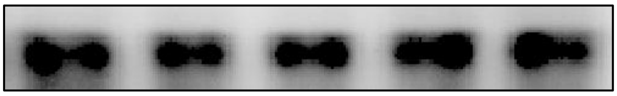

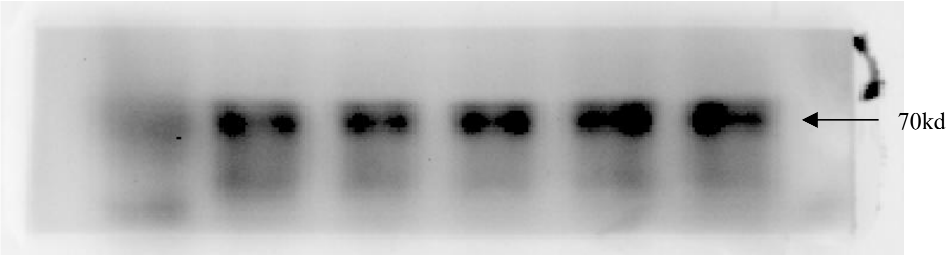

- ② β-actin (NF-κB)

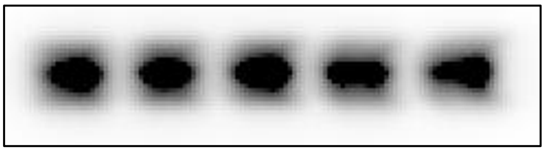

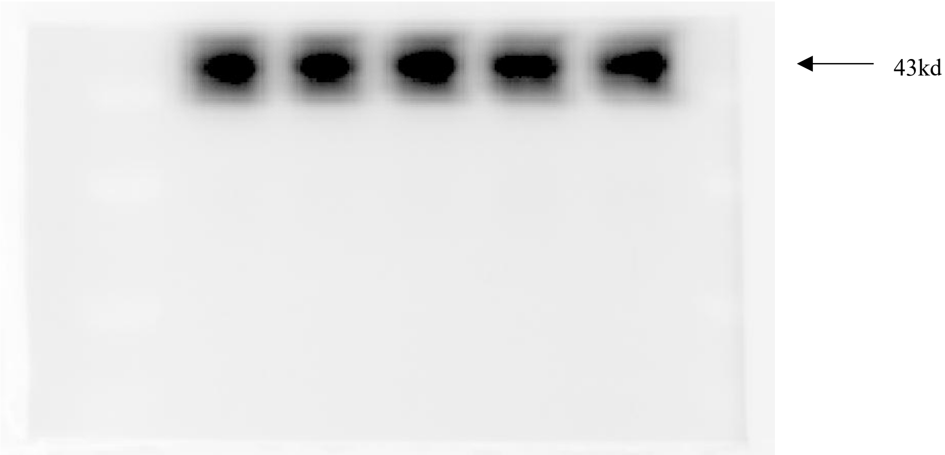

- ② white light (NF-κB)

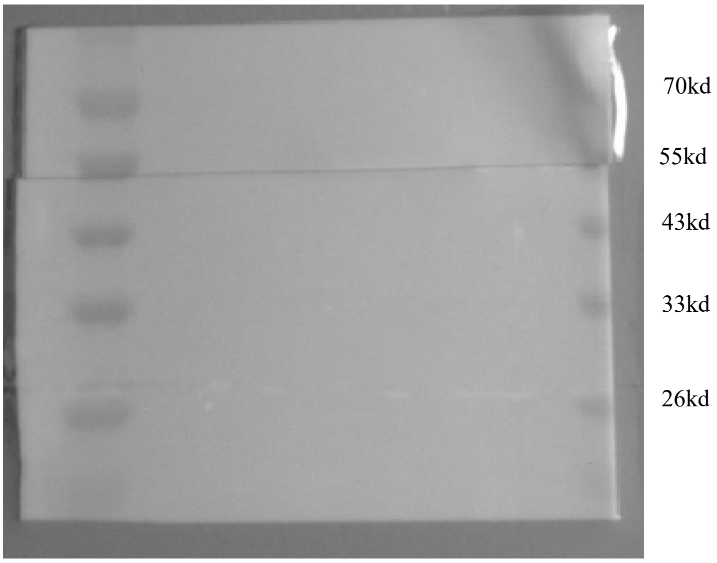

- ③ p-NF-κB

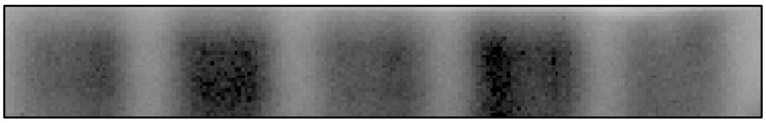

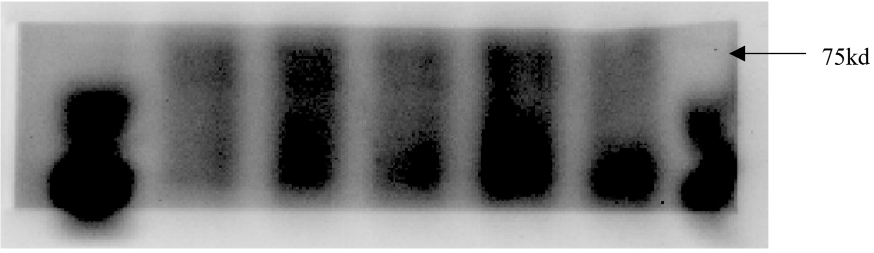

- ③ NF-κB

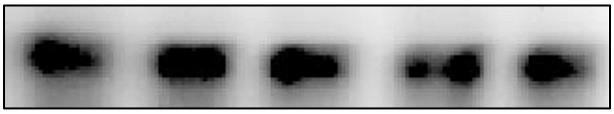

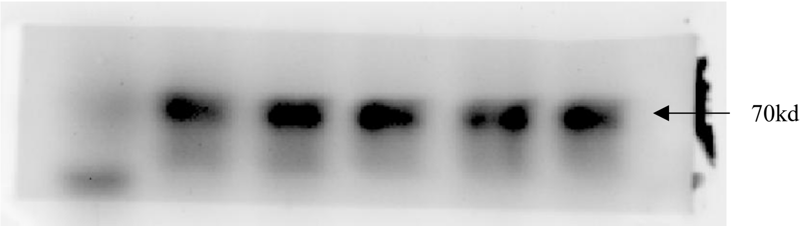

- ③ β-actin (NF-κB)

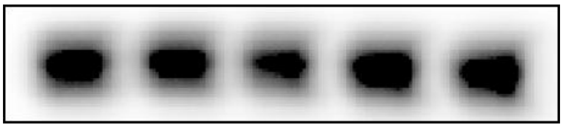

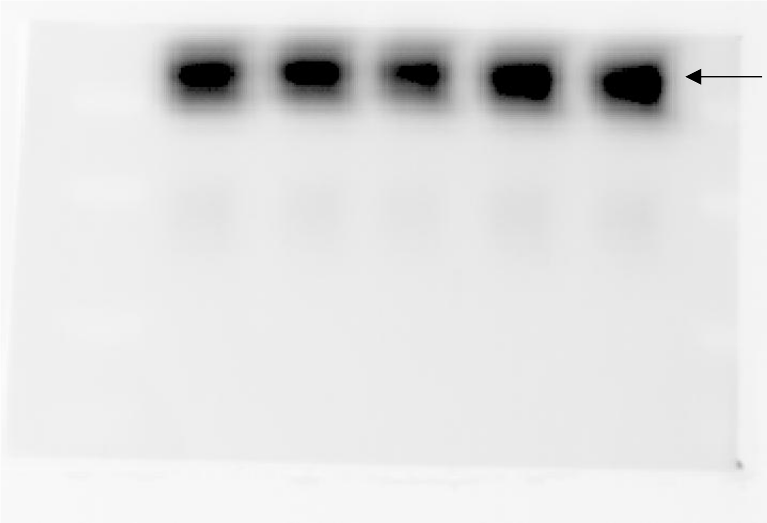

43kd

- ③ white light (NF-κB)

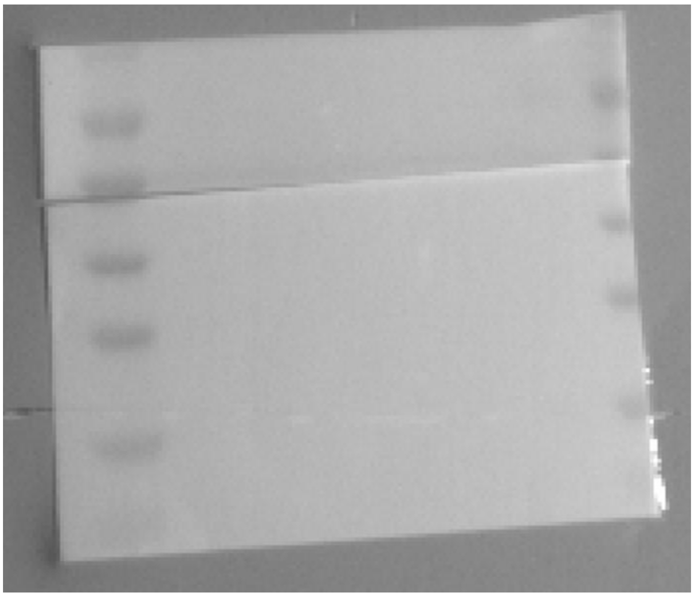

95kd
70kd
55kd
43kd
33kd
26kd

- ④ p-NF-κB

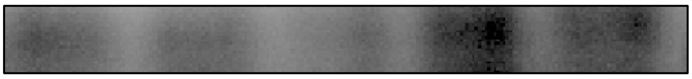

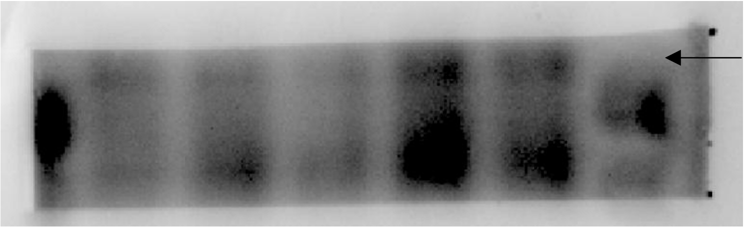

75kd

- ④ NF-κB

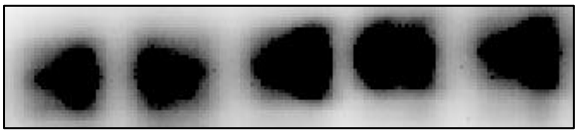

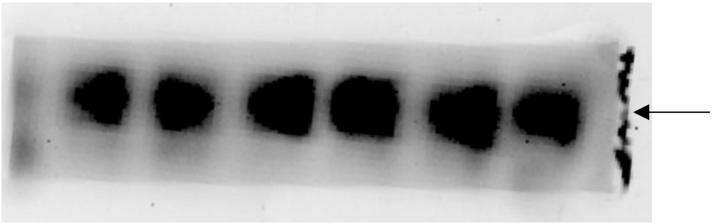

70kd

➢ ④ β-actin (NF-κB)

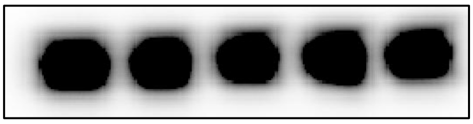

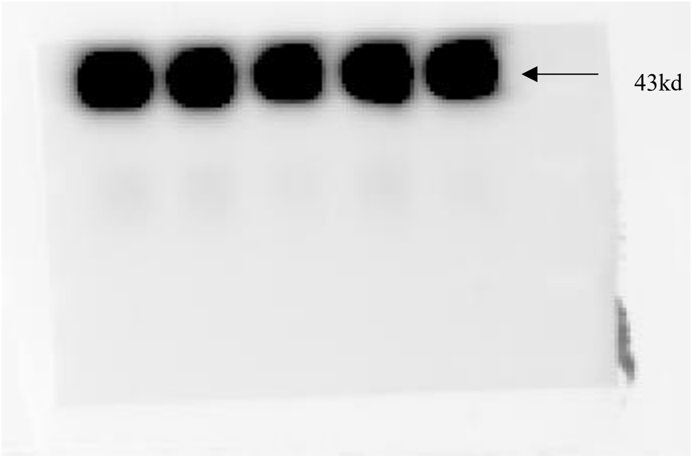

43kd

④ white light (NF-κB)

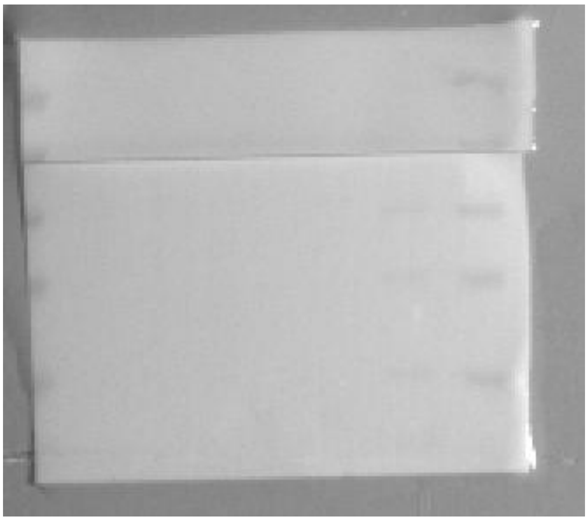

70kd
55kd
43kd
33kd
26kd

- ① Nrf2

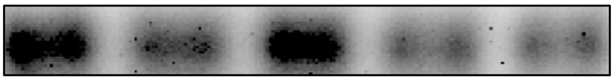

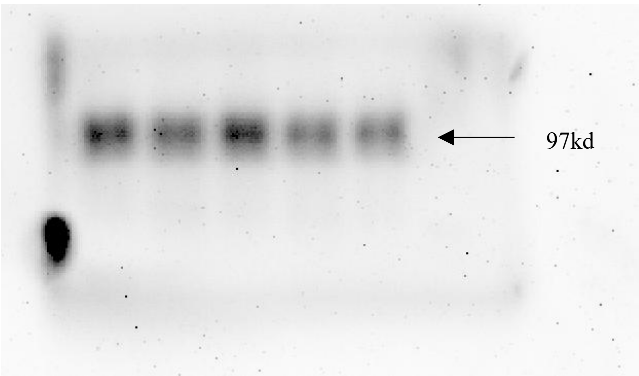

- ① β-actin (Nrf2)

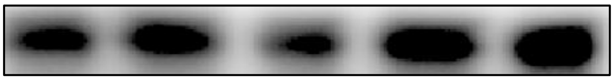

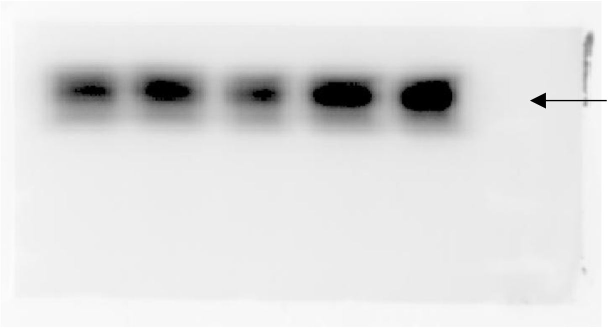

① white light (Nrf2)

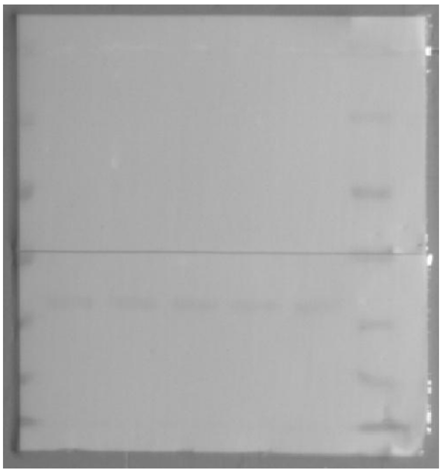

- ② Nrf2

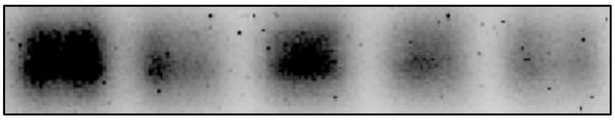

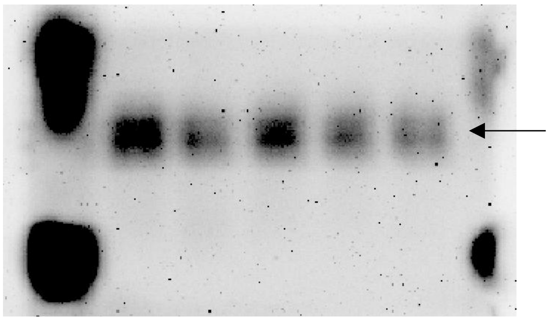

97kd

➢ ② β-actin (Nrf2)

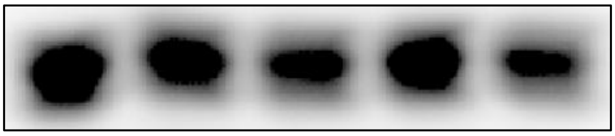

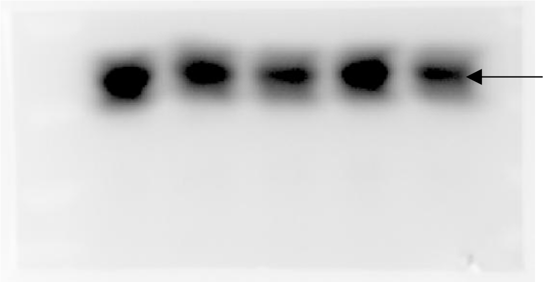

43kd

➢ ② white light (Nrf2)

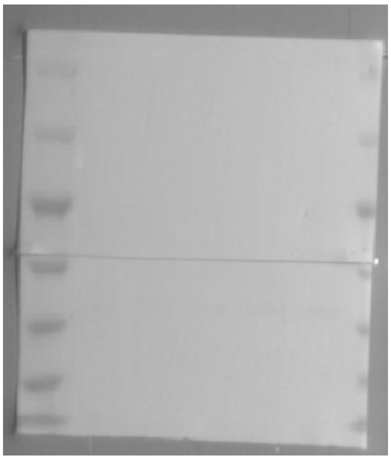

140kd
95kd
70kd
55kd
43kd
33kd
26kd

➢ ③ Nrf2

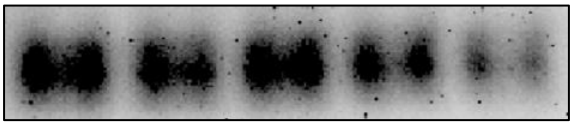

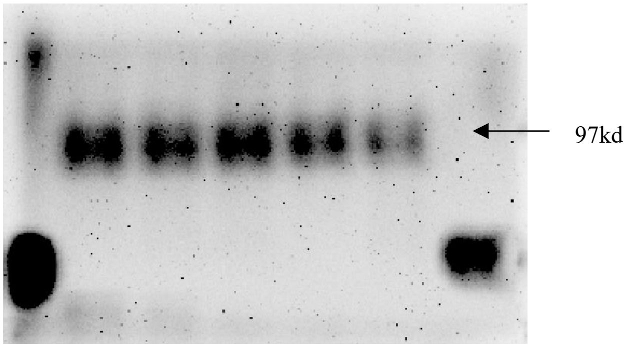

➢ ③ β-actin (Nrf2)

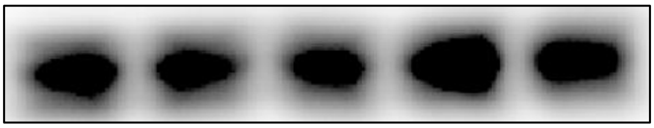

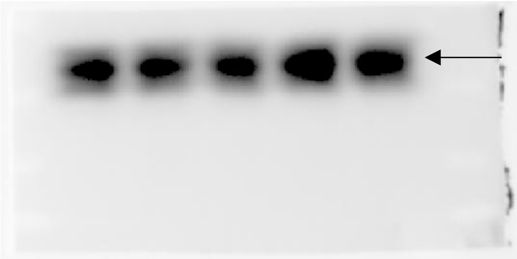

➢ ③ white light (Nrf2)

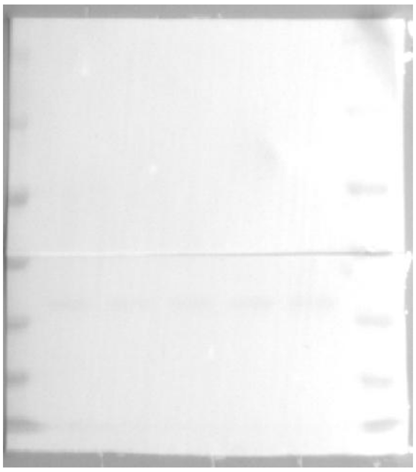

3.FIG6 L

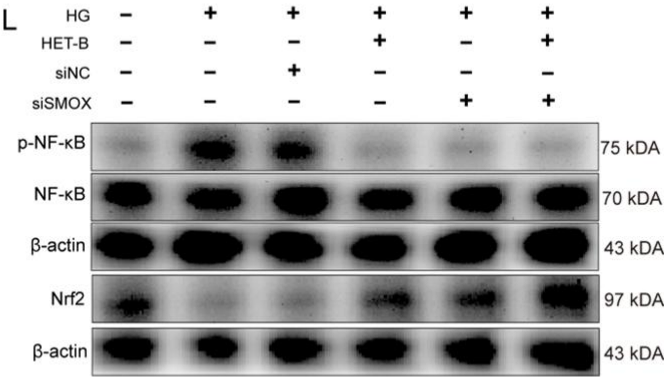

➢ ① P-NF-κB

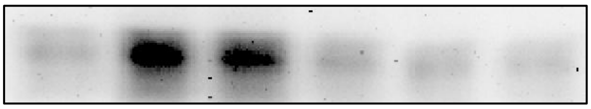

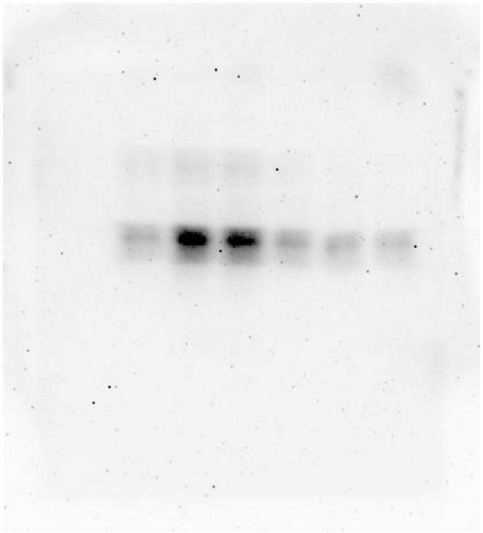

75kd

➢ ① NF-κB

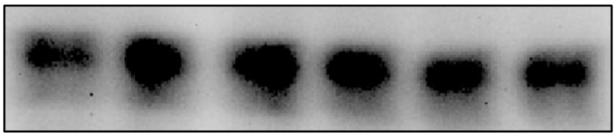

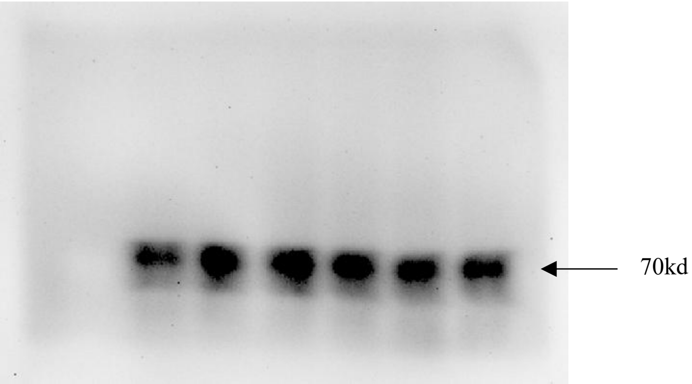

70kd

- ① β-actin (NF-KB)

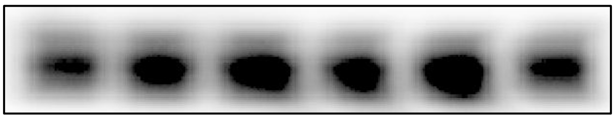

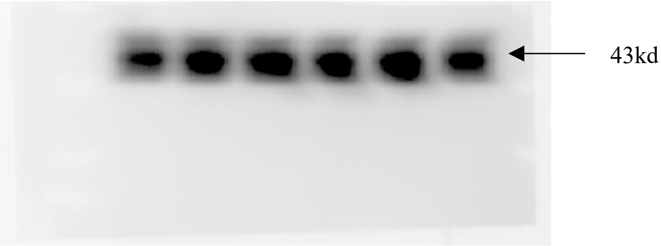

43kd

- ① white light (NF-KB)

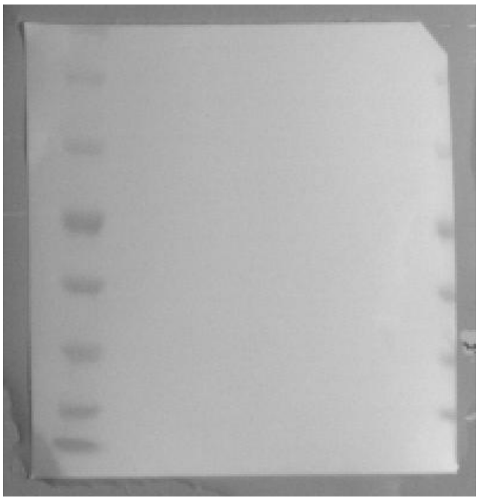

140kd
95kd
70kd
55kd
43kd
33kd

- ② P-NF-κB

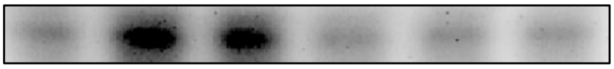

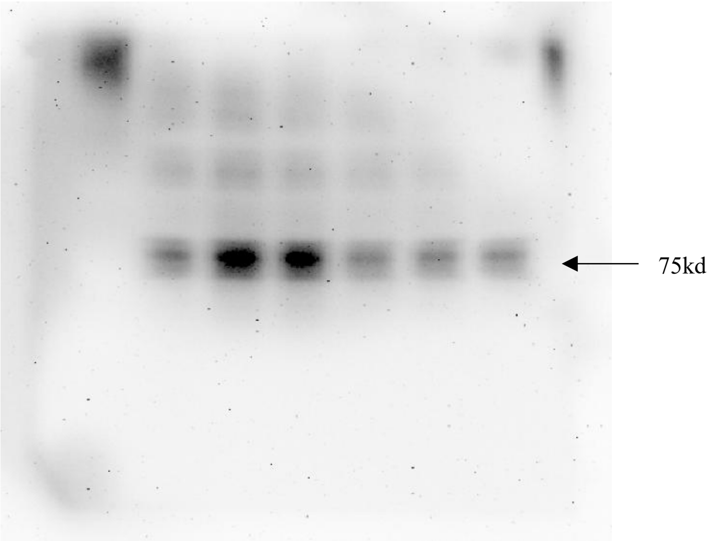

➢ ② NF-κB

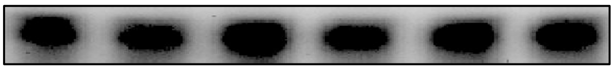

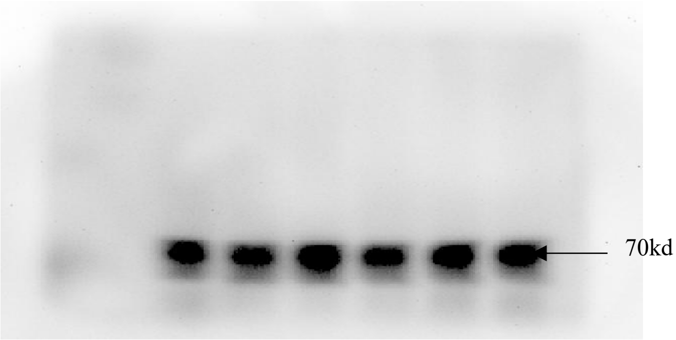

➢ ② β-actin (NF-KB)

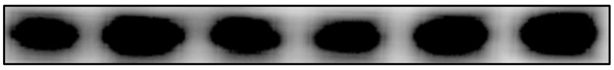

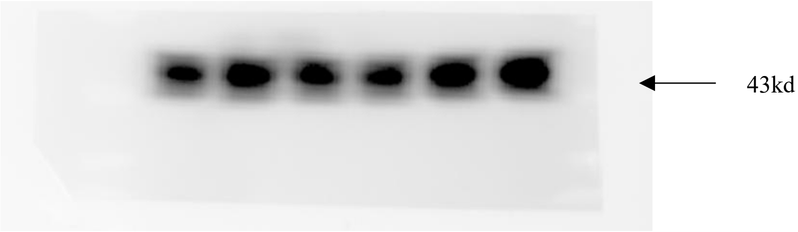

➢ ② White light (NF-KB)

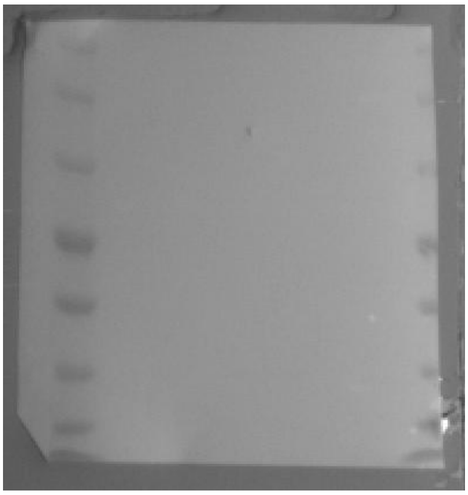

190kd
140kd
95kd
70kd
55kd
43kd
33kd
26kd

- ③ P-NF-κB

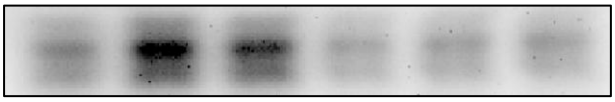

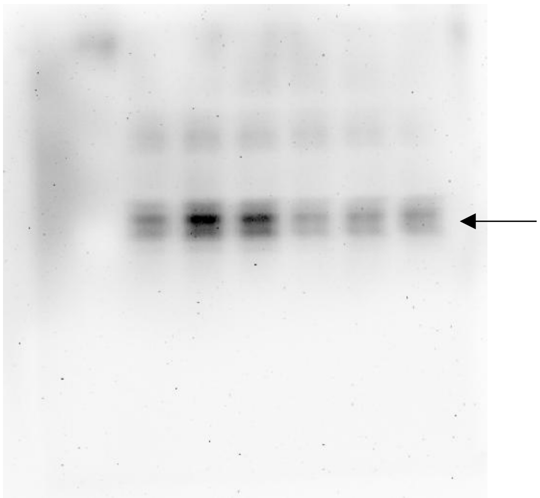

75kd

- ③ NF-κB

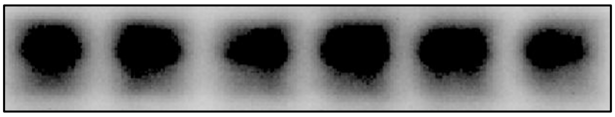

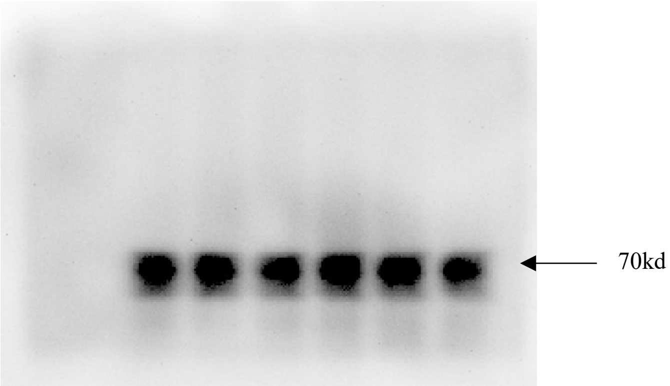

➢ ③ β-actin (NF-KB)

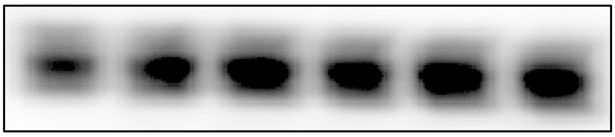

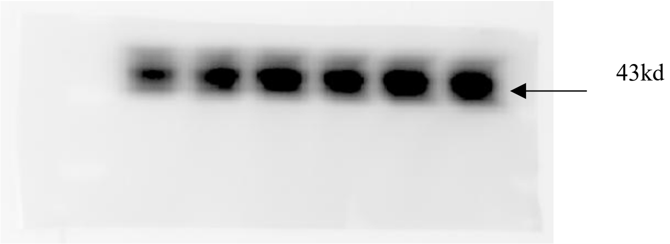

➢ ③ White light (NF-KB)

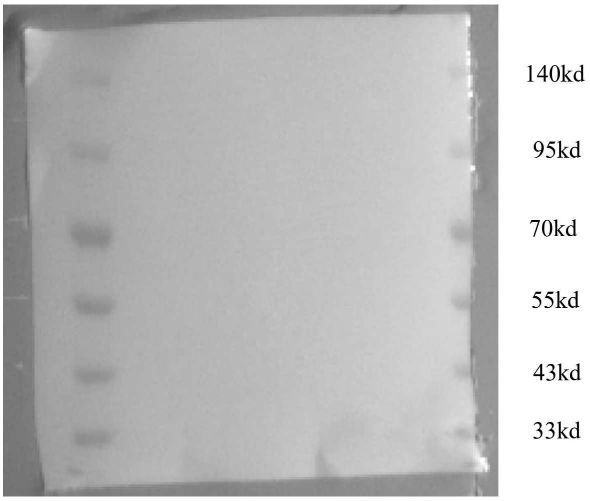

➢ ④ P-NF-κB

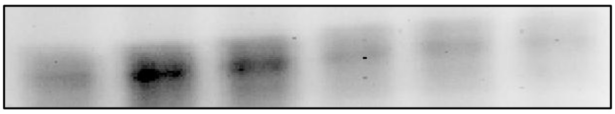

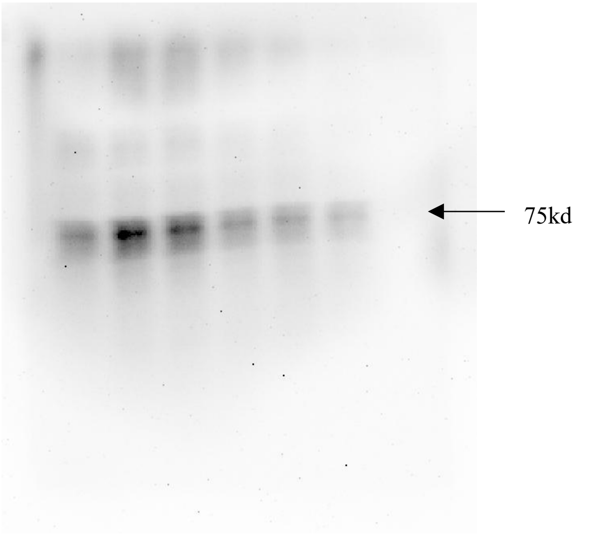

➢ ④ NF-κB

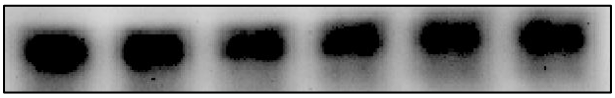

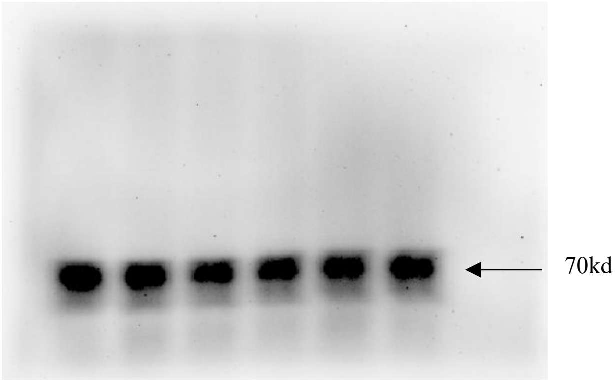

➢ ④ β-actin (NF-KB)

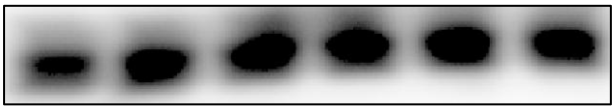

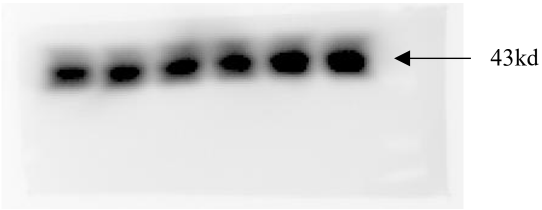

➢ ④ White light (NF-KB)

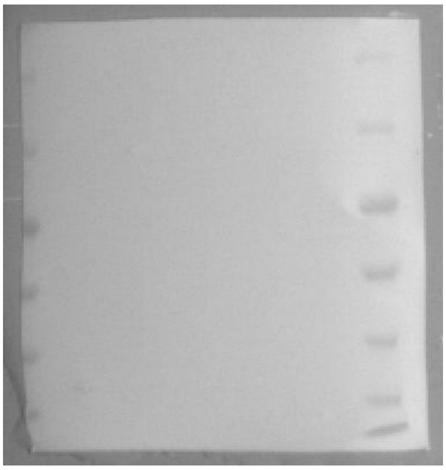

- ① Nrf2

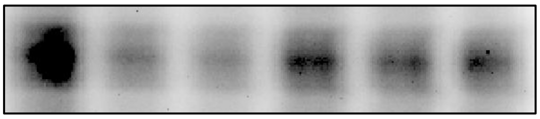

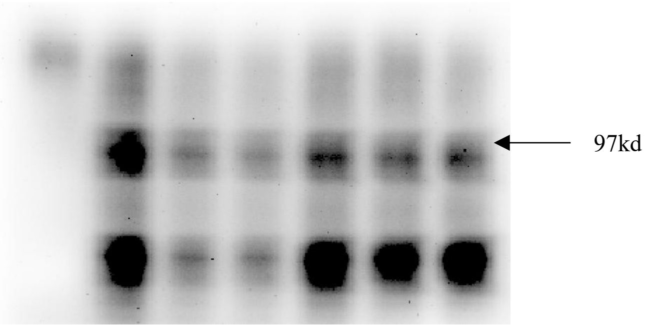

- ① β-actin (Nrf2)

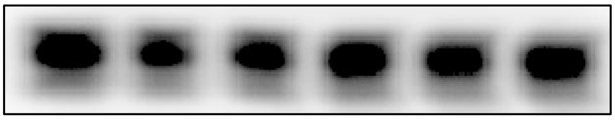

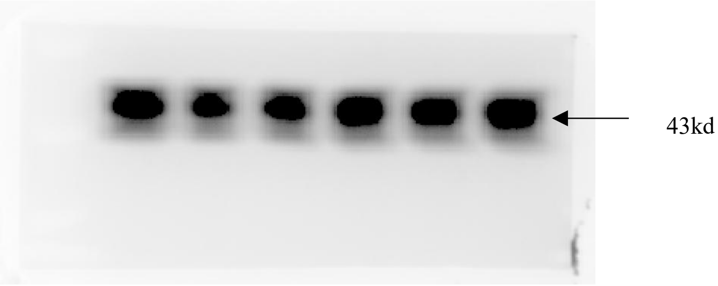

- ① White light (Nrf2)

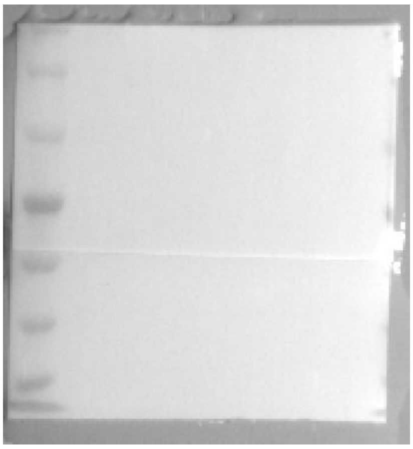

- ② Nrf2

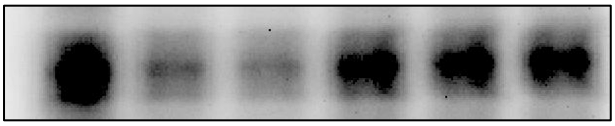

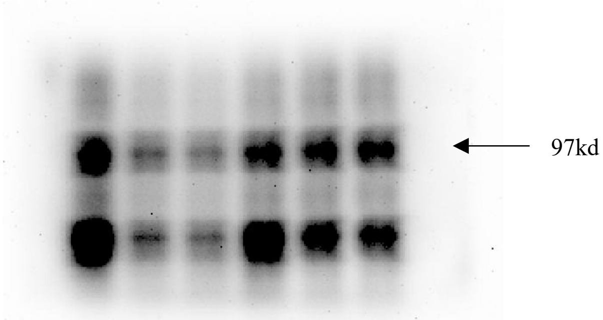

- ② β-actin (Nrf2)

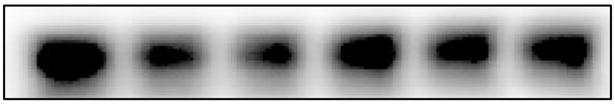

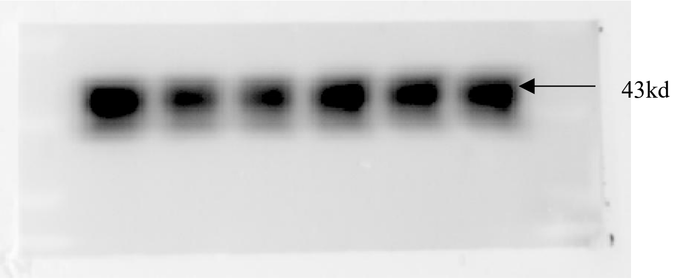

- ② White light (Nrf2)

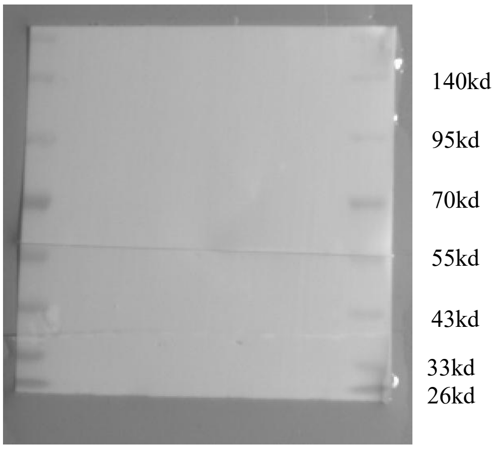

- ③ Nrf2

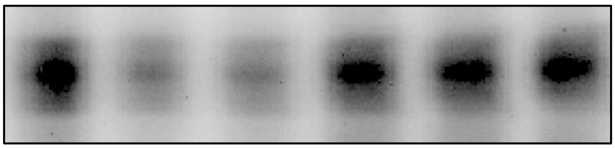

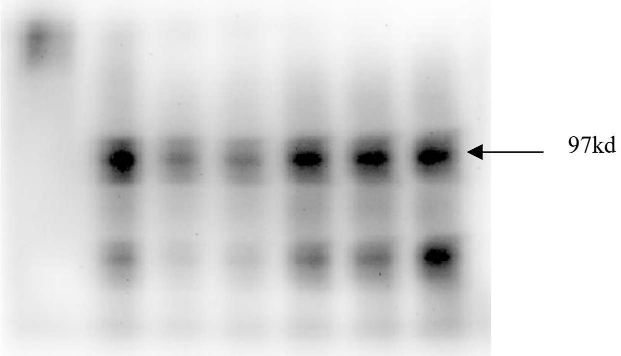

- ③ β-actin (Nrf2)

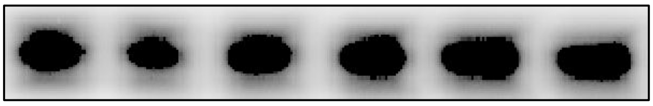

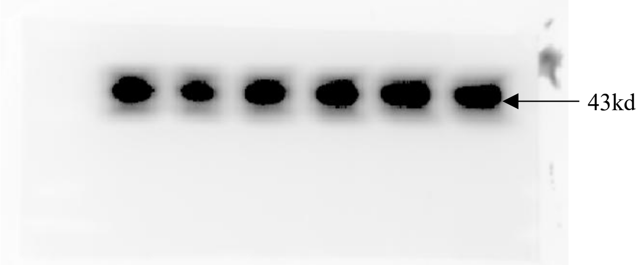

- ③ White light (Nrf2)

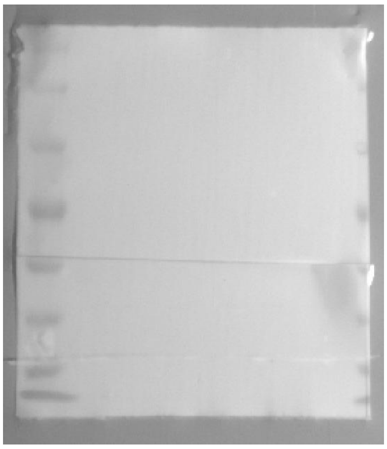

140kd
95kd
70kd
55kd
43kd
33kd
26kd

➢ ④ Nrf2

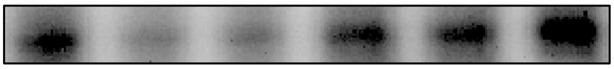

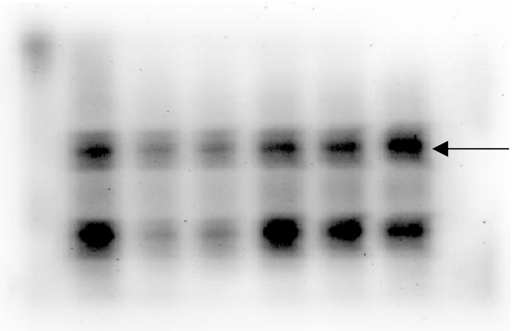

97kd

➢ ④ β-actin (Nrf2)

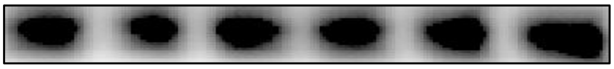

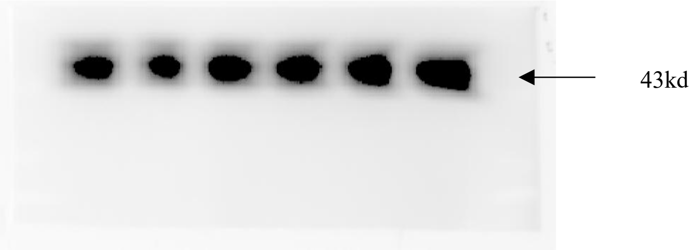

43kd

➢ ④White light (Nrf2)

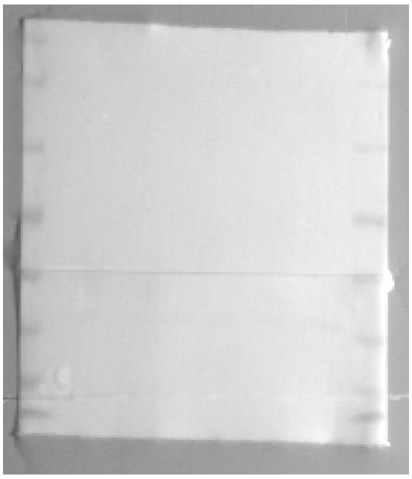

140kd

95kd

70kd

55kd

43kd

33kd
26kd
